# *Akkermansia muciniphila* primes lung-resident antiviral immunity via the gut–lung axis during SARS-CoV-2 infection

**DOI:** 10.3389/fimmu.2026.1762843

**Published:** 2026-02-27

**Authors:** Gi-Cheon Kim, Jun-Soo Do, Sung-Hee Kim, Jong-Hwi Yoon, Jiseon Kim, Donghun Jeon, Emmanuel Hitayezu, Intan Rizki Mauliasari, Naeok Koo, Jeong Jin Kim, Jun-Young Seo, Su-bin Lee, Ki Taek Nam, Kwang Hyun Cha, Ho-Keun Kwon, Je Kyung Seong

**Affiliations:** 1 Department of Microbiology, Institute of Immunology and Immunological Diseases, Yonsei University College of Medicine, Seoul, Republic of Korea; 2 Department of Biomedical Sciences, Graduate School of Medical Science, BK21 Project, Yonsei University College of Medicine, Seoul, Republic of Korea; 3 Systems Biology Research Center, KIST Gangneung Institute of Natural Products, Gangneung, Republic of Korea; 4 Department of Food Science & Aquatic Life Medicine, College of Life Sciences, Gangneung-Wonju National University, Gangneung, Republic of Korea; 5 Laboratory of Developmental Biology and Genomics, Research Institute for Veterinary Science, Seoul National University, Seoul, Republic of Korea; 6 BK21 Project for Creative Veterinary Science Research, College of Veterinary Medicine, Seoul National University, Seoul, Republic of Korea; 7 Korea Mouse Phenotyping Center, Seoul National University, Seoul, Republic of Korea

**Keywords:** *Akkermansia muciniphila*, gut-lung axis, inducible bronchus-associated lymphoid tissue (iBALT), SARS-CoV2 (COVID-19), tissue-resident memory T cells (T_RM_)

## Abstract

**Introduction:**

The gut microbiota plays a central role in shaping systemic immunity and modulating the gut–lung axis, which is crucial during respiratory infections such as COVID-19. SARS-CoV-2 infection is known to disrupt the gut microbiome, but the downstream functional impacts on microbial metabolism and host immune responses remain insufficiently understood.

**Methods:**

Using K18-hACE2 transgenic mice, researchers investigated the effects of SARS-CoV-2 variants (WA and Omicron) on the gut microbiome and host immunity. Microbial composition and functional profiles were assessed post-infection. To test the therapeutic potential of *Akkermansia muciniphila* (*A. muciniphila*), live bacteria were administered prophylactically, and various outcomes were evaluated, including weight loss, lung pathology, immune cell phenotypes, and cytokine production.

**Results:**

In K18-hACE2 transgenic mice infected with SARS-CoV-2, there was a marked reduction in gut microbial diversity, accompanied by a consistent enrichment of *A. muciniphila*. This microbial shift was associated with functional disruptions in key metabolic pathways, particularly those involved in glycosaminoglycan degradation and lipid metabolism, suggesting a broader impact of infection on microbial functionality. Remarkably, prophylactic administration of live *A. muciniphila* prior to infection led to significant protective effects. Treated mice exhibited reduced weight loss and improved lung histopathology compared to untreated controls. Local antiviral immune responses in the lung were notably enhanced without triggering excessive systemic inflammation. Mice receiving *A. muciniphila* also demonstrated elevated production of Th2 and Th17 cytokines, robust expansion of tissue-resident memory T cells, and the formation of inducible bronchus-associated lymphoid tissue (iBALT)—all indicative of potentiated mucosal immunity. These findings highlight a functional role for *A. muciniphila* not only as a microbial signature of COVID-19-associated dysbiosis but also as an active modulator of host immune responses during respiratory viral infections.

**Discussion:**

These findings position *A. muciniphila* as both a biomarker of COVID-19-related gut dysbiosis and a potent live biotherapeutic candidate for respiratory infections. Its ability to enhance mucosal immune responses through gut–lung axis modulation highlights its promise in prophylactic strategies against viral respiratory diseases, including SARS-CoV-2.

## Introduction

The gut microbiota, a dynamic and diverse microbial ecosystem, is essential for maintaining host health by regulating immune responses, preserving epithelial barrier integrity, and ensuring metabolic homeostasis (1). Among its multifaceted roles, the gut microbiota is a cornerstone of the gut–lung axis, a bidirectional communication pathway connecting the gastrointestinal and respiratory systems via microbial metabolites, immune cell trafficking, and systemic signaling pathways (2, 3). Through this axis, the intestinal microbiota can shape distal pulmonary immune responses—for example, short-chain fatty acids (SCFAs) produced by commensal bacteria have been shown to calibrate antiviral and inflammatory pathways in influenza infection (4, 5). These insights emphasize that microbial ecology in the gut can profoundly influence respiratory immunity. In the context of COVID-19, the gut–lung axis has garnered increasing attention as a critical determinant of disease severity and recovery (6–8).

Severe acute respiratory syndrome coronavirus 2 (SARS-CoV-2), the virus responsible for COVID-19, has been shown to profoundly disrupt gut microbiota composition. Human studies have reported decreased microbial diversity, depletion of beneficial taxa such as *Faecalibacterium* and *Bifidobacterium*, and enrichment of pro-inflammatory or opportunistic pathogens (8)—for instance, a decline in short-chain fatty acid (SCFA)-producing bacteria like *Faecalibacterium prausnitzii* has been associated with elevated systemic inflammation in severe COVID-19 cases. Recent clinical studies have further demonstrated that COVID-19-associated gut dysbiosis is linked to adverse pulmonary outcomes and prolonged symptoms, underscoring the clinical importance of this axis (7). Additionally, dysbiosis in COVID-19 patients has been observed to persist beyond viral clearance, indicating a prolonged impact on immune regulation and lung recovery (7, 9). These changes compromise gut barrier integrity, promote microbial translocation into the bloodstream, and exacerbate systemic inflammation and cytokine storms, both hallmarks of severe COVID-19 (8, 10–12). Alterations in gut-derived metabolites, including SCFAs, further contribute to pulmonary immune dysregulation, emphasizing the gut microbiota’s critical role in respiratory health (3, 13). The clinical relevance of understanding COVID-19-induced gut dysbiosis lies in its potential for therapeutic intervention. Microbiome-targeted strategies, such as SCFA supplementation, probiotics, and fecal microbiota transplantation (FMT), have shown promise in restoring microbial balance and modulating immune responses in other infectious diseases (13–15). Applying these strategies to COVID-19, particularly by targeting the gut–lung axis, offers a novel approach to mitigate systemic inflammation and improve respiratory outcomes (8, 16, 17).

Importantly, accumulating clinical and preclinical evidence indicates that SARS-CoV-2 variants differ markedly not only in transmissibility and tissue tropism but also in their immunopathological profiles (18)—for example, the ancestral Wuhan (WA) strain is known to induce severe pulmonary inflammation and mortality in K18-hACE2 mice, whereas Omicron and other variants cause a milder disease with attenuated cytokine responses and reduced viral burden in the lungs despite robust replication in the upper airways (18–20). These phenotypic differences may stem from divergent interactions with the host immune system, including differences in interferon sensitivity, viral entry efficiency, and epithelial tropism, and could plausibly extend to their impact on the gut microbiota (21, 22). Given that gut-resident microbes are highly sensitive to systemic immune tone, cytokine milieu, and tissue-specific inflammation, each SARS-CoV-2 variant may impart distinct signatures of gut dysbiosis and microbial metabolite alterations (6). Such strain-specific effects on host–microbe interactions could influence not only acute disease severity but also post-infection recovery and long-term immune remodeling. However, these effects remain poorly understood in both clinical and experimental settings.

Despite these insights, human studies are limited in capturing the longitudinal, variant-specific, and age-dependent dynamics of gut microbiota changes during SARS-CoV-2 infection. To address this gap, animal models are essential. The K18-hACE2 transgenic mouse model is particularly valuable because it reproduces key features of human COVID-19 pathology and allows the controlled dissection of host–microbe interactions in a temporal manner (23–25). Therefore, we employed it to investigate the temporal effects of infection with both ancestral (WA) and Omicron (Omi) SARS-CoV-2 variants on gut microbiota composition. Our longitudinal profiling revealed consistent patterns of dysbiosis across both variants, including reduced microbial diversity, loss of commensal taxa, and a marked increase in the relative abundance of *Akkermansia muciniphila*—a mucin-degrading bacterium known to influence host immunity via the gut–lung axis. Intriguingly, *A. muciniphila* levels were positively correlated with infection-associated physiological decline in mice and were similarly elevated in multiple independent cohorts of human COVID-19 patients. This convergence across species led us to hypothesize that *A. muciniphila* expansion may represent a host-compensatory response with potential immunomodulatory effects. To test this, we conducted mono-colonization experiments in antibiotic-pretreated mice, revealing that *A. muciniphila* stably engrafted at physiologically relevant levels and attenuated infection-associated weight and temperature loss while enhancing localized pulmonary immune responses. These findings not only validate the relevance of animal models for dissecting SARS-CoV-2–microbiota interactions but also highlight *A. muciniphila* as a promising microbial candidate for the therapeutic modulation of host responses to respiratory viral infections.

## Materials and methods

### Mice

**Figure 1:**
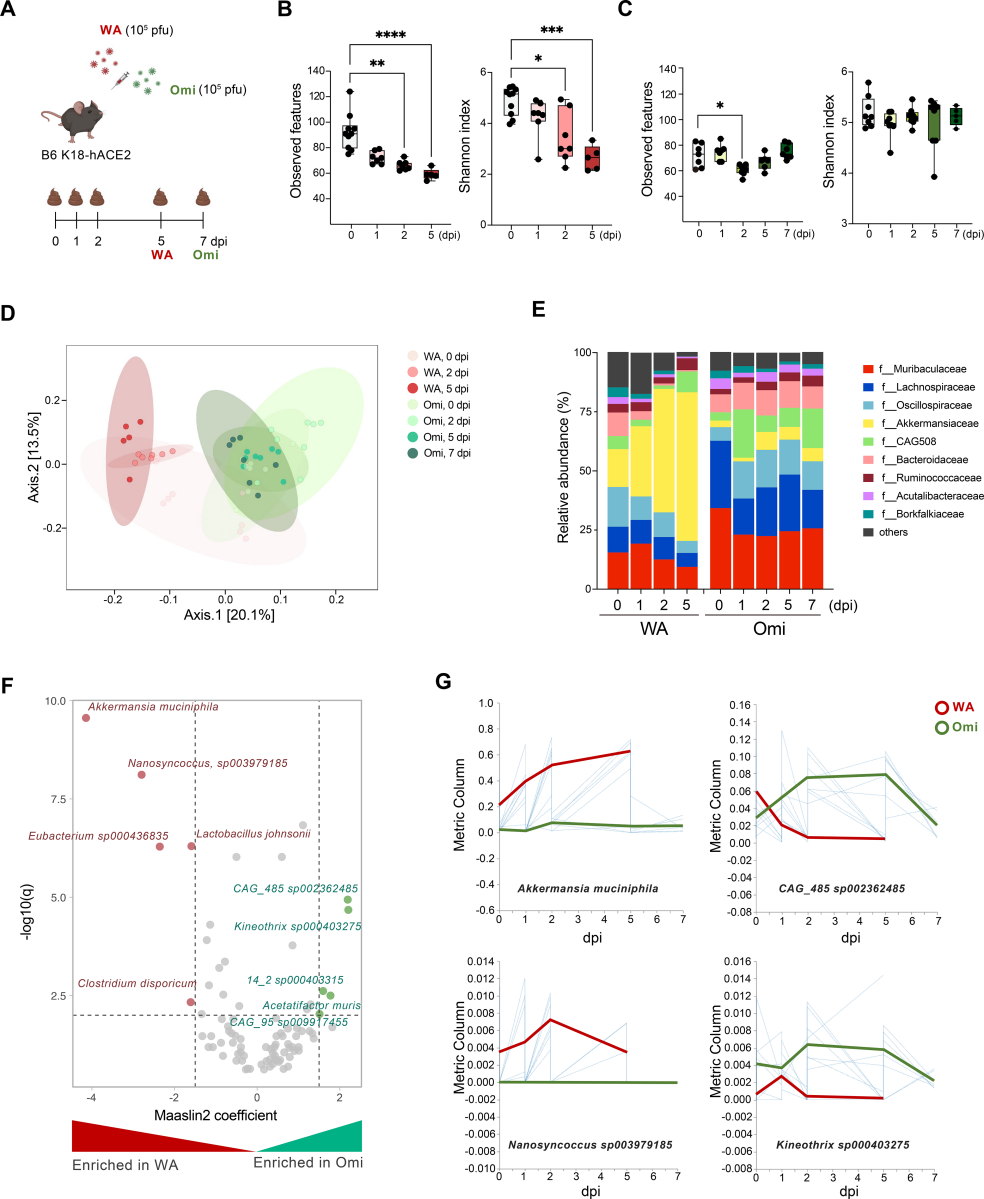
Gut microbiome alterations in mice by SARS-CoV-2 infection. **(A)** Study design for two types (WA and Omi) of SARS-CoV-2 infection. Fecal samples were collected based on the indicated timeline, and the number of mice per group for 16S rRNA sequencing was *n* = 5–8, as indicated by dots in the figure. Microbiome analysis was performed on all surviving animals at each time point. Alpha diversity (observed features and Shannon index) of **(B)** WA-infected mice and **(C)** Omi-infected mice. **(D)** Principal coordinate plot of bacterial compositions at 0, 2, and 5 dpi for WA infection and at 0, 2, 5, and 7 dpi for Omi infection (unweighted UniFrac distance). **(E)** Taxa bar plot of feces microbiome after WA or Omi infection at the family level. Bars represent the average microbial composition across individuals at each time point. **(F)** Volcano plot of Maaslin2 multivariate analysis results from SARS-CoV-2-infected mice to identify differential abundant species between WA and Omi infection. Virus types and dpi were included as fixed effects and mice ID as a random effect. X-axis indicates Maaslin2 coefficient, and the Y-axis is log10(FDR-corrected *p*-values). **(G)** Volatility plots for the two most significant taxa in each group from Maaslin2 analysis based on WA and Omi infection time. The global mean and importance values were calculated by q2-longitudinal plugin in QIIME2. Statistical significance values in box plots were determined using Kruskal–Wallis test with the comparison of 0 dpi group. *p < 0.05; **p < 0.01; ***p < 0.001; ****p < 0.0001.

**Figure 2:**
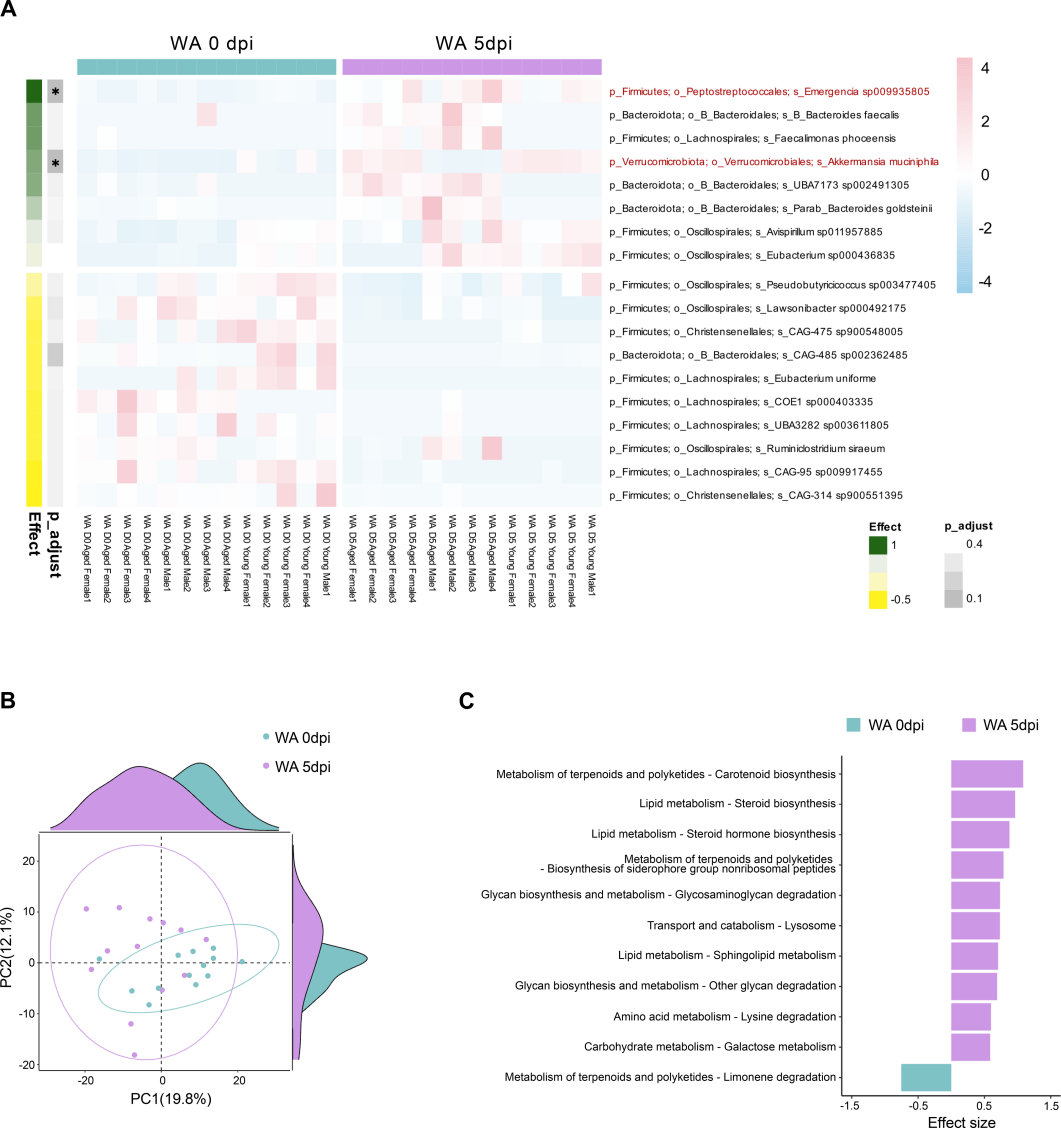
Differentially abundant gut microbiota and predicted gene function before (0 dpi) and after (5 dpi) WA infection. ALDEx2 was used to perform pairwise differential abundance tests between groups and to calculate the expected standardized effect sizes and false discovery rate for the paired data from merged WA-infected aged and young mice experiments. **(A)** Scaled heatmap of 18 differentially abundant gut microbiota across samples in the species level (p, phylum; o, order; s, species). The abundant bacteria were aligned based on ALDEx2 effect sizes, and the colors represent the z-scores of each species’ relative abundance. The significant taxa were indicated in red font color. **(B)** PCA plot of the relative abundance profiles of PICRUSt2-predicted microbial functional pathways enriched in WA 0 and 5 dpi groups. **(C)** ALDEx2 effect size of PICRUSt2-predicted KEGG pathways before and after WA infection. Statistical significance values were determined using Wilcoxon rank-sum test (*adjusted *p* < 0.05).

**Figure 3:**
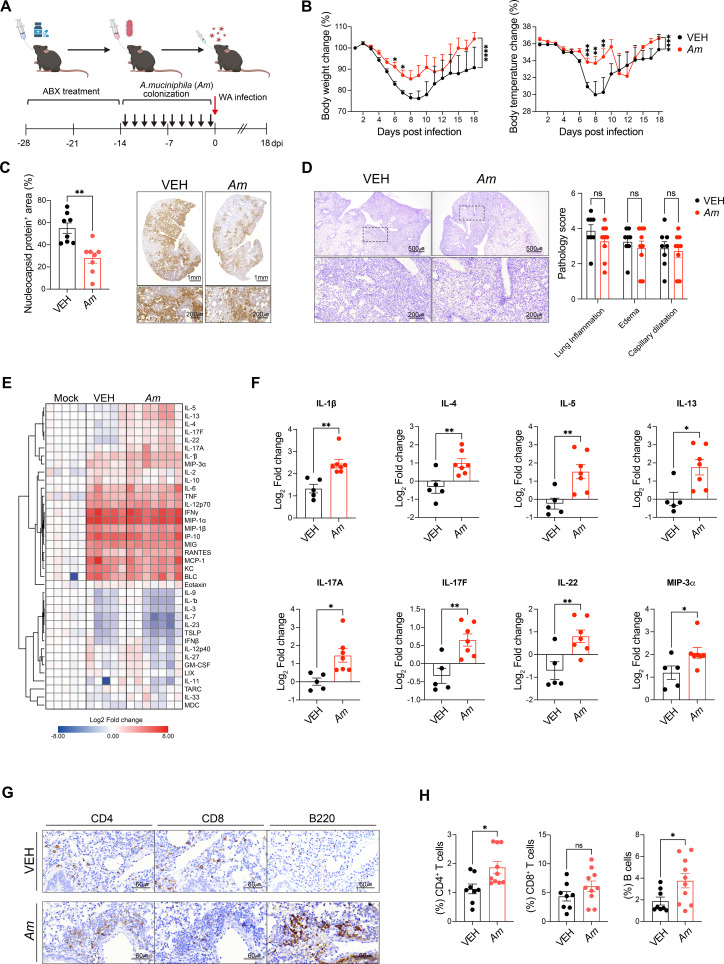
*Akkermansia muciniphila* reduces COVID-19-induced pathology and modulates immune responses. **(A)** Schematic of the experimental design. **(B)** Body weight (left) and temperature change (right) after SARS-CoV-2 infection. Data points represent daily measurements (*n* = 8–10 per group). Statistical significance was calculated using two-way ANOVA. *p < 0.05; **p < 0.01; ***p < 0.001; ****p < 0.0001. **(C)** Quantification of nucleocapsid protein-positive areas in lung sections (left) and representative immunohistochemistry images of lung tissue (right) from both groups. **(D)** Histopathological analysis of lung (*n* = 6–8 mice per group) and representative H&E-stained lung sections. **(E)** Heatmap showing cytokine profile changes in the lungs of mock, vehicle, and *A. muciniphila*-treated mice. Mock treatment refers to animals receiving sterile PBS without bacteria or virus, serving as baseline controls. **(F)** Fold change of multiple cytokines of lung, including IL-5, IL-13, IL-4, IL-17F, IL-22, IL-17A, IL-1β, and MIP-3α in the lung tissue. Cytokine levels are represented as log2 fold changes compared to vehicle treatment. **(G)** The immunohistochemical staining results for immune markers (CD4, CD8, B220) in lung tissue sections are shown. **(H)** Proportion and positive cells of CD4, CD8, and B cells in the lung. Statistical significance was determined using unpaired *t*-tests. *p < 0.05; **p < 0.01; ns, not significant.

Adult male K18-hACE2 transgenic mice (B6.Cg-Tg(K18-ACE2)2Prlmn/J, JAX stock no. 034860) were purchased from Jackson Laboratories and used for all experiments. Adult mice (8–10 weeks old) were used for standard infection and treatment experiments, while aged mice (77 weeks old) were used for aging-associated comparisons. The animal procedures were conducted in a biosafety level 3 (BSL3) facility in compliance with the Public Health Service Policy on Humane Care and Use of Laboratory Animals. The experimental protocol was approved by the Institutional Animal Care and Use Committee (IACUC) of Department of Laboratory Animal Resources of Yonsei University College of Medicine, accredited by the Association for Assessment and Accreditation of Laboratory Animal Care (AAALAC) International (protocol no. 001071). For infection, the mice were anesthetized with a mixture of ketamine and xylazine and intranasally inoculated with SARS-CoV-2. The infection dose was determined based on the experimental objective: 1 × 10^5^ PFU was used for longitudinal microbiome profiling (**Figures 1**, **2**), and 1 × 10^2^ PFU was used for survival and therapeutic efficacy studies (**Figure 3**).

### Cells and viruses

African green monkey kidney epithelial Vero E6 cells were cultured in Dulbecco’s modified Eagle’s medium (Hyclone) supplemented with 10% fetal bovine serum (Hyclone) and 1% penicillin–streptomycin (Hyclone). The cell line was maintained at 37°C and in a 5% CO_2_ atmosphere. The cells were authenticated and routinely tested negative for mycoplasma contamination. The original Wuhan strain of SARS-CoV-2 (accession number: NCCP43326/Korea) and the SARS-CoV-2 Omicron variant (BA.1, accession number: NCCP43408/Korea) were obtained from the Korea Centers for Disease Control and Prevention. The virus was produced by infecting Vero E6 cells cultured in DMEM containing 2% FBS for 2 to 3 days (MOI of 0.5). Supernatant containing the virus was collected and clarified by centrifugation (4,000 rpm for 15 min) before storage at –80°C. All experiments used viral stocks with ≤3 passages. Titer of viral stock was determined by plaque assay. All work relating to SARS-CoV-2 virus was conducted in the BSL-3 facility of Avison Biomedical Research Center in accordance with institutional biosafety committee regulations.

### Plaque assay

Infectious SARS-CoV-2 plaque-forming units were quantified by plaque titration on Vero E6 cells. At 1 day before infection, the cells were seeded in 12-well plates at a density of 1 × 10^5^ cells/mL. After washing once with DMEM without FBS, the cells were inoculated with viruses serially diluted in DMEM containing 2% FBS at 1:10 dilution for 1 h at 37°C. The inoculum was removed, and the cells were washed with DMEM without FBS and subsequently overlaid with a 1:1 mix of 2% sea-plaque agarose (Lonza) and 2× DMEM supplemented with 2% FBS and 1% penicillin–streptomycin. After 48–72 h of incubation at 37°C, the cells were fixed with 4% paraformaldehyde (Biosesang) at 4°C overnight and visualized by crystal violet staining. The number of plaques was counted, and the virus titer was calculated. For infected mice, mouse tissues were chopped in phosphate-buffered saline (PBS) and stored at −80°C until further processing. Viruses were extracted from the chopped tissues by three freeze–thawing cycles. The virus supernatant was collected and clarified by centrifugation and 0.2-µm filter. The virus titers in the supernatant were quantified using the plaque assay method described previously.

### Preparation of *Akkermansia muciniphila* stocks

*Akkermansia muciniphila* (ATCC BA-835) cultures were prepared according to a modified protocol adapted from a previous study (26). The bacteria were cultivated in Gifu Anaerobic Medium (GAM; MB Cell, Seoul, South Korea), supplemented with 0.2% mucin (porcine stomach mucin type III, Sigma Aldrich, USA). A frozen glycerol stock stored at -80°C was initially streaked onto a GAM–mucin agar plate and incubated at 37°C for 24 h. A single colony from this plate was subsequently selected for expansion in a 2-L culture. Following large-scale cultivation, bacterial cells were harvested, transferred to sealed 50-mL tubes, and centrifuged at 3,000 rpm for 30 min at 4°C. The pellets were then resuspended in fresh medium and stored as 50% glycerol stocks with a concentration of 6.06 × 10^10^ CFU/mL. Colony-forming units (CFU) were quantified by serial dilution of one stock, plating on GAM–mucin agar, and counting colonies after 72 h of incubation at 37 °C. All procedures, except centrifugation, were conducted under strict anaerobic conditions in an anaerobic chamber (Coy Lab Products) with a gas mixture of 85% N_2_, 5% H_2_, and 10% CO_2_.

### Antibiotic pretreatment and *Akkermansia muciniphila* treatment

To deplete the gut microbiota, mice were treated with a broad-spectrum antibiotic cocktail administered via oral gavage for 7 days, followed by an additional 7 days of antibiotic treatment provided in their drinking water. The antibiotic mixture for gavage included ampicillin (1 mg/mL), neomycin (1 mg/mL), metronidazole (1 mg/mL), and vancomycin (0.5 mg/mL) (Sigma Aldrich). After completing the 7-day gavage treatment, the same antibiotic cocktail was supplied in the drinking water for another 7 days. Following the 14-day antibiotic regimen, drinking water was replaced with sterile water for 24 h to ensure the clearance of residual antibiotics. For *Akkermansia muciniphila* treatment, the mice were orally gavaged with 1 × 10^8^ CFU of *A. muciniphila* suspended in 200 μL of sterile PBS every other day for a total of 10 treatments. The control mice received an equivalent volume of sterile PBS without bacteria. To confirm successful colonization, fecal samples were collected and analyzed for the presence of *A. muciniphila* by qPCR targeting the *A. muciniphila*-specific 16S rRNA genes as described previously (27). Once colonization was verified, the mice were used for SARS-CoV-2 infection experiments or other study protocols.

### Preparation of single-cell suspension

For the isolation of primary immune cells, lungs and spleens were harvested at 7 days post-infection. Lung tissues were minced into small pieces and digested in RPMI 1640 medium containing 1 mg/mL collagenase type V (Sigma Aldrich) and 20 U/mL DNase I (Sigma Aldrich) for 30 min at 37 °C with gentle stirring. After digestion, the cell suspension was passed through a 40-µm cell strainer to remove debris and obtain single-cell suspensions. Red blood cells (RBCs) were lysed using RBC lysis buffer (BioLegend). For spleen cell isolation, the spleens were mechanically dissociated and filtered through a 40-µm strainer to obtain single-cell suspensions. RBC lysis was performed with RBC lysis buffer (BioLegend).

### Flow cytometric analysis

Single-cell suspensions were washed with ice-cold PBS before being resuspended in 40 μL of PBS for surface marker staining. The following antibodies were used for surface staining: Live Dead (Fixable aqua), CD45 (BUV395), MHCII (BUV496), CD8 (BUV496), NK1.1 (BUV563), CD44 (BUV615), F4/80 (BUV661), CD62L (BUV737), TCRβ (BV421), CD11c (BV480), LY6G (BV570), TCRβ (BV605), CD4 (BV650), TCRβ (BV650), Ly6C (BV786), CD69 (PE), CD103 (PE-Dazzle594), CD19-biotin (PE-Cy5), TCRγδ (APC), CD8 (APC-Cy7), and CD11b (SparkBlue550). For intracellular staining of transcription factors and cytokines, the cells were first surface-stained, followed by fixation and permeabilization using Fixation/Permeabilization Buffer (eBioscience). The intracellular markers used for staining included IFN-γ (BUV737), GRANZYME B (Pacific Blue), TNFα (BB700), IL-2 (PE), PERFORIN (PE-Dazzle594), and FOXP3 (AF488). Tetramer staining was performed by, first, staining the cells with the SARS-CoV-2 peptide-MHC class I tetramer (NIH Tetramer Core Facility), followed by subsequent staining with surface and intracellular markers. Detailed information regarding the antibodies used, including clone numbers and manufacturers, is listed in **Supplementary Table S1**. The gating strategies for identifying immune cell populations are presented in **Supplementary Figure S4**. Data acquisition was carried out using a SONY ID7000 flow cytometer, and analysis was performed using FlowJo software (Tree Star). For dimensionality reduction and visualization of immune cell populations, the UMAP (Uniform Manifold Approximation and Projection) algorithm was employed.

### Cytokine measurement

Lung homogenates and serum were prepared at 7 days post-infection, and cytokine levels were measured using LEGENDplex™ Mouse Th Cytokine Panel (cat. no. 741043), Mouse Cytokine Panel 2 (cat. no. 740134), and Mouse Proinflammatory Chemokine Panel (cat. no. 740007) (BioLegend, San Diego, CA, USA) according to the manufacturer’s instructions. Data were analyzed using a Luminex 200 system and LEGENDplex software.

### Histopathology and immunohistochemistry

Lung tissues were fixed in 10% neutral-buffered formalin (Sigma, St. Louis, MO, USA) for 24 h and embedded in paraffin, sectioned to a thickness of 4 μm, and processed for hematoxylin and eosin (H&E) staining or immunohistochemistry (IHC). For H&E staining, slides were deparaffinized, immersed in 0.1% Mayer’s hematoxylin for 10 min, and counterstained with 0.5% eosin. The slides were dehydrated through a graded ethanol series (50%, 70%, 95%, and 100%) and mounted using a mounting solution (Thermo Fisher Scientific, Waltham, MA, USA). The stained slides were evaluated by veterinary pathologists. The stained slides were independently analyzed by three veterinary pathologists.

For IHC, deparaffinized slides were rehydrated through xylene, 100%, 95%, and 70% ethanol, followed by distilled water. Antigen retrieval was performed using a pH 6.0 citrate buffer (Dako S1699, Agilent Technologies, Santa Clara, CA, USA) under high temperature in a pressure cooker, followed by cooling on ice for 1 h. To block endogenous peroxidase activity, the slides were incubated with 3% H_2_O_2_ in PBS for 30 min, followed by treatment with M.O. reagent (Vector Laboratories, Burlingame, CA, USA) for 1 hour. The slides were then incubated overnight at 4°C with primary antibodies, including SARS-CoV-2 N protein (NB100-56576, Novus; 40143-MM08, Sino Biological), CD4 (25229s, Cell Signaling), CD8 (98941, Cell Signaling), F4/80 (ab6640, Abcam), LY-6G/LY-6C (ab2557, Abcam), and CD45R(B220) (ab64100, Abcam). Afterward, the slides were treated with a protein-blocking solution (Dako) for 1 h, followed by incubation with an HRP-conjugated secondary antibody (Dako) for 15 min. The IHC signal was developed using DAB substrate (Dako), and nuclear counterstaining was performed with Mayer’s hematoxylin. For immunofluorescence staining, Alexa 488-conjugated anti-mouse, anti-rat, and anti-goat IgG antibodies, along with Cy3-conjugated anti-rabbit IgG antibodies, were applied. Images were acquired using a Zeiss LSM980 confocal microscope. Areas of bronchus-associated lymphoid tissue (BALT), characterized by B220-positive follicles, were identified as PTPRC (B220)-positive regions (28).

### Weight change and body temperature monitoring

Mouse weight change and body temperature were monitored daily for 21 days post-infection. Weight change was calculated as a percentage of the baseline value recorded on the day of infection, and body temperature was measured using an implantable programmable temperature transponder (IP55-300, BMDS).

### Fecal DNA extraction

Fecal DNA extraction from mice was performed on all surviving animals at each time point for microbiome analysis. The total genomic DNA from fecal samples was extracted using the phenol-chloroform extraction method. Briefly, fecal samples were suspended in 300 μL of TE buffer and subjected to mechanical disruption using a bead beater. The disrupted samples were then treated with an equal volume of phenol/chloroform/alcohol (25:24:1) solution and 10% SDS solution centrifuged at 12,000 × *g* for 10 min at 4°C. The aqueous phase containing DNA was transferred to a new tube and precipitated by adding 1 volume of 100% ethanol. The precipitated DNA was pelleted by centrifugation at 12,000 × *g* for 10 min at 4°C, washed with 70% ethanol, and resuspended in nuclease-free water.

### 16S rRNA amplicon sequencing and data processing

The 16S rRNA gene was amplified using Illumina-adapted universal primers, 341F and 805R, targeting the V3–V4 region. Polymerase chain reaction (PCR) was performed with ExTaq polymerase (Takara Bio, Kusatsu, Japan), starting with an initial denaturation at 98°C for 3 min, followed by 25 cycles of denaturation at 95°C for 30 s, annealing at 55°C for 30 s, and extension at 72°C for 30 s, with a final extension at 72°C for 5 min. The PCR products were purified and quantified using AMPure XT beads (Beckman Coulter Genomics, Danvers, MA, USA) and Qubit dsDNA High-Sensitivity Assay Kit (Invitrogen; Carlsbad, CA, USA), respectively. The normalized amplicons were prepared for sequencing with Nextera XT DNA Library Preparation Kit (Illumina, San Diego, CA, USA) in combination with Nextera XT Index Kit. Sequencing was carried out on the MiSeq platform using the MiSeq Reagent Kit v3 (600 cycles) (Illumina). Sequencing reads were demultiplexed, and paired-end reads were processed by QIIME2 pipeline (version 2024.5) (29). DADA2 workflow was applied for the quality trimming, denoising, and filtering against chimeric PCR artifacts (30). The resulting exact amplicon sequence variants (ASVs) were then assigned taxonomy using the Greengenes 2 reference database (2022.10) through q2-greengenes plugin within QIIME2 (31). The 16S rRNA gene amplicon datasets obtained by this research were deposited in National Center for Biotechnology Information (NCBI) under accession numbers.

### Microbiome data statistical analysis

Alpha diversity indices such as observed features and Shannon were calculated using QIIME2 platform. For the diversity comparison among groups, Kruskal–Wallis test was used. Unweighted UniFrac metrics was used to assess beta diversity, and principal coordinate plot was generated by MicrobiomeAnalyst (32). To statistically evaluate the differences in microbial community composition (beta-diversity) between groups, permutational multivariate analysis of variance (PERMANOVA) was performed with 999 permutations using unweighted UniFrac distance. The R package “edgeR” was used to extract differentially abundant genus for volcano plots, and the volcano plots were produced using VolcaNoseR online platform (33, 34). For differential abundance analysis, raw counts were first filtered to exclude low-abundance taxa: samples with fewer than 8,000 total reads and genera with a relative abundance of less than 0.1% were removed. The remaining data were normalized using the trimmed mean of M-values (TMM) method. Differential abundance was determined using a generalized linear model (GLM), and *P*-values were adjusted for multiple testing using the Benjamini–Hochberg false discovery rate (FDR) approach (FDR < 0.05). To identify differentially abundant microbes considering multivariable associations, we used the R package “MaAsLin2” (Microbiome Multivariable Associations with Linear Models) with two fixed effects (virus types and dpi) and one random effect (mice ID) (35). Longitudinal analysis was performed using the q2-longitudinal plugin and volatility analysis was used to determine how the bacterial abundance changed over the virus infection period (36). The R package “ALDEx2” was used to analyze significant differences in gut microbiota composition and predicted metabolic pathways between groups for merged (aged and young) and paired samples, employing the Wilcoxon rank-sum test (37). Functional pathway prediction was performed using PICRUSt2 based on the Kyoto Encyclopedia of Genes and Genomes (KEGG) database using the 16S rRNA gene sequencing data (38). The ALDEx2 results were visualized as heatmap, PCA plot, and bar chart of effect sizes using the R package, including “ALDEx2” and “pheatmap”.

### Human fecal sample analysis

To investigate changes in *Akkermansia* abundance following SARS-CoV-2 infection in human patients, we utilized public data from the Sequence Read Archive (SRA) and National Genomics Data Center (NGDC). We analyzed over 700 samples from five independent Bioprojects (PRJNA624223, PRJEB43555, PRJNA650244, PRJNA758913, and CRA003945) and extracted the relative abundance percentage values of “Bacteria” and “*Akkermansia muciniphila*” using the SRA Taxonomy Analysis Tool (STAT) (**Supplementary Table S2**). To minimize technical variability and batch effects across different SRA datasets, we applied stringent metadata filtering, including only samples with clearly defined clinical history and infection status. COVID-19 disease severity was classified according to each study’s definitions: asymptomatic and mild cases were designated as severity I, moderate cases showing pneumonia symptoms as severity II, and severe and critical cases as severity III. Considering that *Akkermansia*, unlike other common gut microbiota, tends to be characterized by carrier and non-carrier status, we first identified *Akkermansia* presence in each sample and then conducted further analyses specifically on *Akkermansia* carriers.

### Statistical analysis

All data were expressed as means ± standard error of the mean. Statistical comparisons between groups were performed using unpaired *t*-test. Correlations between microbiota abundance and weight or temperature change were analyzed using Spearman’s correlation. Statistical significance was set at *p <*0.05. All statistical analyses were conducted using GraphPad Prism (GraphPad Software).

## Results

### The impact of WA and Omi infection on gut microbiota composition

To investigate the effects of SARS-CoV-2 variants (WA and Omi) on gut microbiota dynamics, we infected B6 K18-hACE2 mice with each variant (10^5^ PFU) and collected fecal samples at multiple time points. Due to the high lethality of WA infection, samples were analyzed up to 5 days post-infection (dpi), while Omi-infected mice, which survived longer, provided samples up to 7 dpi (**Figure 1A**). Microbial diversity analyses revealed clear differences in the impact of the two variants on gut microbial composition. In WA-infected mice, species richness, measured by observed features, declined significantly at 2 dpi and remained low at 5 dpi. By contrast, Omi-infected mice exhibited only a transient decrease at 2 dpi, followed by recovery at 5 dpi (**Figures 1B, C**). Similarly, the Shannon diversity index, which captures both richness and evenness, showed a sustained reduction in WA-infected mice but remained stable in Omi-infected mice over the same period. These findings indicate that WA infection induces more profound and persistent loss of microbial diversity, whereas Omi infection allows partial recovery, reflecting differential host–microbe interactions. Beta diversity analysis further highlighted the divergence between the two variants. Principal coordinate analysis (PCoA) of beta diversity demonstrated a significant shift in microbial community structure in WA-infected mice at 5 dpi, indicating persistent dysbiosis (**Figure 1D**). In contrast, the microbiota composition of Omi-infected mice remained relatively stable, with clustering patterns similar to pre-infection states. This suggests that WA infection causes more severe and lasting disruption of gut microbial homeostasis compared to Omi infection. Taxonomic analysis revealed variant-specific changes in microbial composition. *Akkermansia muciniphila* (*A. muciniphila*), a mucin-degrading bacterium implicated in intestinal barrier modulation and immune regulation, was enriched in both WA- and Omi-infected mice. However, its relative abundance was significantly higher in WA-infected mice, where it dominated the microbial community (**Figure 1E**). This disproportionate increase in *A. muciniphila* in WA-infected mice suggests a potential role in the exacerbation of gut dysbiosis and host immune dysregulation. Maaslin2 analysis further identified additional taxa enriched in WA-infected mice, including *Nanosyncoccus* sp*003979185*, *Eubacterium* sp*000436835*, and *Lactobacillus johnsonii*. Conversely, Omi-infected mice were enriched with taxa such as *CAG_485* sp*002362485*, *Kineothrix* sp*000403275*, and *Acetatifactor muris* (**Figure 1F**). Volatility analysis of the most significantly altered taxa highlighted the dynamic nature of gut microbiota changes during infection. In WA-infected mice, microbial disruption persisted, with sustained decreases in beneficial taxa and enrichment of dysbiosis-associated species. In contrast, Omi-infected mice exhibited transient disruptions followed by recovery, suggesting the resilience of the gut microbiota to Omi infection (**Figure 1G**). Collectively, these findings highlight the greater impact of WA infection on gut microbiota composition compared to Omi infection, with WA inducing more profound and persistent dysbiosis. The significant enrichment of *A. muciniphila* in WA-infected mice underscores its potential role as a key modulator of the gut–lung axis, contributing to the pathophysiology of SARS-CoV-2 infection.

### Gut microbiota and functional alterations associated with WA infection

We sought to determine whether WA infection induces specific gut microbiota and functional changes. We analyzed microbial composition and predicted metabolic pathways in both young and aged K18-hACE2 mice since aging is key determinant for COVID-19 severity (39). Consistent with prior findings, aged mice exhibited higher baseline microbial diversity compared to young mice (40, 41), which remained unchanged following WA infection (**Supplementary Figures S1A–C**). Despite this, a significant increase in *Akkermansia* abundance was observed in both age groups at 5 days post-infection (dpi), reaffirming its enrichment during WA infection (**Figure 2A**; **Supplementary Figure S1D**). To identify microbial taxa specific to WA infection, we performed ALDEx2 pairwise analysis by combining datasets from young and aged mice. This analysis revealed the significant enrichment of *Akkermansia* and *Emergencia* after WA infection (*p*-adjust < 0.05), along with increases in *Bacteroides faecalis* and *Faecalimonas phoceensis*. In contrast, the *Christensenellales* order, previously abundant in pre-infection samples, showed a marked reduction at 5 dpi (**Figure 2A**). We next analyzed potential functional shifts in the gut microbiome. We used PICRUSt2 to predict microbial metabolic pathways from the same dataset. Principal component analysis (PCA) based on the inferred metagenomic profiles indicated a separation in predicted functional potential before and after WA infection (**Figure 2B**). While most pathways showed minimal changes (effect size < 1), glycosaminoglycan degradation—a hallmark function of mucin-degrading bacteria like *Akkermansia*—was significantly increased at 5 dpi (**Figure 2C**). Furthermore, the predicted abundance of several lipid-related pathways, including carotenoid biosynthesis, steroid biosynthesis, steroid hormone biosynthesis, and sphingolipid metabolism, were inferred to be significantly enriched following WA infection (**Figure 2C**). These findings align with the lipid-encapsulated nature of SARS-CoV-2 virions and reported lipid metabolism alterations in infected patients (42, 43). These results together suggest that WA infection induces specific changes in gut microbial composition and predict functional potential, characterized by the dominance of *Akkermansia* and elevated lipid-related pathways. These alterations likely reflect complex host–microbe interactions during SARS-CoV-2 infection and may contribute to the pathophysiology of severe COVID-19.

### SARS-CoV-2 infection reshapes the gut microbiota with *Akkermansia* as a disease-associated indicator

We first investigated gut microbiota changes associated with SARS-CoV-2 pathogenesis by assessing correlations between microbial taxa and physiological parameters—namely, weight loss and body temperature reduction—following WA strain infection in mice. Among the microbial taxa identified, members of the Akkermansiaceae (*Akkermansia muciniphila*) and the Eubacterium fissicatena groups showed significant positive correlations with both disease-related parameters, suggesting a potential link between their expansion and infection-induced physiological decline (**Supplementary Figures S2A–C**). Conversely, several taxa within the Lachnospiraceae and Ruminococcaceae families were negatively associated with weight and temperature loss, suggesting a potentially protective role in the context of infection. To assess whether these patterns were conserved in humans, we analyzed gut microbiota profiles from five independent COVID-19 cohorts (7, 11, 44–46). *A. muciniphila* was consistently detected at higher frequency and relative abundance in COVID-19 patients compared to healthy controls across all datasets (**Supplementary Figures S2D, E**). Although data were integrated from multiple studies, the consistent enrichment of *Akkermansia* across independent cohorts suggests a robust biological signal that outweighs study-specific batch effects. When stratified by disease severity, individuals with mild disease (severity I) exhibited significantly higher *A. muciniphila* levels than controls, whereas those with moderate to severe disease (severity II and III) showed greater inter-individual variability and a trend toward reduced abundance (**Supplementary Figure S2F**). These findings suggest that *A. muciniphila* expansion may represent a host-compensatory or protective response to SARS-CoV-2 infection, prompting us to examine its functional role in disease modulation.

### *A. muciniphila* mono-colonization ameliorates SARS-CoV-2-induced pathophysiology via localized immune modulation

To directly test the potential immunomodulatory role of *A. muciniphila* in SARS-CoV2 infection, we utilized a pseudo-germ-free mouse model generated by broad-spectrum antibiotic (ABX) pretreatment followed by mono-colonization with *A. muciniphila*. Notably, infection severity—measured by weight and survival rate—was comparable between ABX-treated infected mice and untreated infected controls, suggesting that microbiota depletion alone had minimal impact on disease outcomes (data not shown). This established a clean baseline to evaluate the specific contributions of *A. muciniphila*. Following mono-colonization (1 × 10^9^ CFU), the mice were challenged with SARS-CoV-2 (WA strain, 1 × 10² PFU) (**Figure 3A**). Stool analysis confirmed successful colonization, with *A. muciniphila* levels in mono-colonized mice reaching approximately fivefold higher than those in SPF animals, suggesting that mono-colonization led to robust and physiologically relevant engraftment of *A. muciniphila* (**Supplementary Figure S3A**). Remarkably, *A. muciniphila* pre-treatment significantly mitigated weight and temperature loss compared to vehicle-treated controls (**Figure 3B**). Moreover, lung tissue analysis revealed a reduction in SARS-CoV-2 nucleocapsid-positive cells in colonized mice (**Figure 3C**), although viral titers remained unchanged (**Supplementary Figure S3B**), indicating that *A. muciniphila* limits local viral spread without affecting total replication levels. Notably, despite these protective effects, histopathological assessment of the lung showed no significant differences in lung inflammation, edema, or capillary dilatation between the VEH and *A. muciniphila* groups (**Figure 3D**), suggesting that the observed benefits were not associated with overt tissue pathology. Immunological profiling of the lung microenvironment demonstrated that *A. muciniphila* treatment selectively enhanced local cytokine responses. Th2-associated cytokines (IL-4, IL-5, and IL-13) and Th17-associated cytokines (IL-1β, IL-17A, IL-17F, IL-22, and MIP-3α) were significantly upregulated in lung homogenates, whereas systemic cytokine levels in the serum remained unaltered (**Figures 3E, F**; **Supplementary Figure S3C**). Flow cytometry and histological analysis further showed increased frequencies of lung-resident CD4^+^ T cells, CD8^+^ T cells, and B cells in *A. muciniphila*-colonized mice, alongside elevated macrophages and neutrophils (**Figures 3G, H**; **Supplementary Figures S3D, E**). These changes were accompanied by the formation of organized lymphoid aggregates resembling inducible bronchus-associated lymphoid tissue (iBALT). Collectively, these data indicate that *A. muciniphila* fosters localized antiviral immunity and lymphoid tissue organization, potentially contributing to the containment of SARS-CoV-2 pathogenesis without triggering systemic inflammation.

### *Akkermansia muciniphila* enhances lung T-cell responses and tissue-resident memory formation

**Figure 4:**
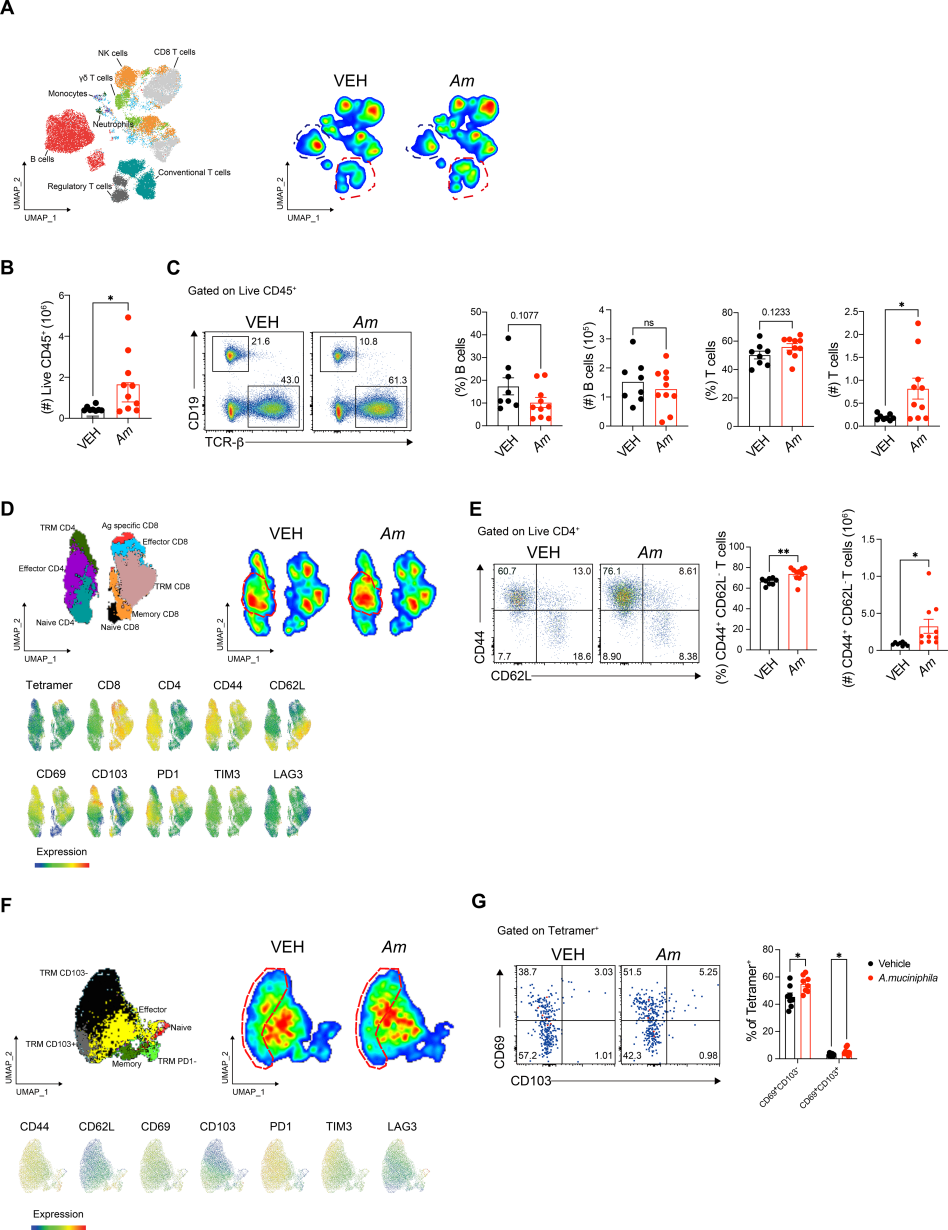
*Akkermansia muciniphila* pre-treatment alters immune cell populations and enhances CD4^+^ and CD8^+^ T-cell responses in the lungs. **(A)** UMAP analysis showing the clustering of immune cell populations from lung tissue, including NK cells, CD8^+^ T cells, γδ T cells, monocytes, neutrophils, B cells, regulatory T cells, and conventional T cells (left) and density plots of immune cells from lung tissue comparing vehicle-treated and **(A)** *muciniphila*-treated groups (right). **(B)** Quantification of total immune cell counts in the lung. **(C)** Proportion and number of T cells and B cells in the lung. **(D)** UMAP projection of T cells showing the distribution of T-cell subsets in vehicle-treated and *A. muciniphila*-treated lungs (left) and density plots of T cells highlighting the increased presence of effector T cells in the *A. muciniphila*-treated group (right). **(E)** Quantification of CD44^+^ CD62L^-^ effector CD4 T cells. **(F)** UMAP analysis of CD8^+^ T cells specific to SARS-CoV-2 using tetramer staining, showing the distinct clustering of tissue-resident memory (T_RM_) cells (left) and density plots of CD8^+^ T_RM_ cells in the lung tissue of vehicle-treated and *A. muciniphila*-treated mice (right), highlighting the increased population of CD69^+^ CD103^+^ T_RM_ cells in the *A. muciniphila*-treated group. **(G)** Quantification of CD69^+^ CD103^+^ T_RM_ cells in COVID-19-specific CD8^+^ T cells. Statistical significance values were determined using unpaired *t*-tests. *p < 0.05; **p < 0.01; ns, not significant.

To investigate the immune changes in the lung mediated by *A. muciniphila* pre-treatment, we analyzed various immune cell populations in lung tissue and compared them to vehicle-treated controls (**Supplementary Figure S4**). UMAP analysis revealed diverse immune cell populations, including NK cells, CD8^+^ T cells, γδ T cells, monocytes, neutrophils, B cells, and both regulatory and conventional T cells (**Figure 4A**). Consistent with earlier histological observations (**Figure 3**), *A. muciniphila* pre-treatment significantly increased T cell abundance while reducing B cell proportions across lung samples (**Figure 4A**). Notably, this was accompanied by a significant overall increase in the total number of lung immune cells compared to vehicle-treated mice (**Figure 4B**). A further subset analysis revealed a pronounced shift toward T cell dominance. While the proportion of B cells decreased, CD4^+^ T cells increased significantly following *A. muciniphila* treatment (**Figures 4A, C**). In contrast, no significant changes were observed in NK cells, γδ T cells, or myeloid cell populations (**Supplementary Figures S5A, B**). Focusing on CD4^+^ T cell subsets, we identified a significant increase in effector CD4^+^ T cells (CD44^+^ CD62L^-^) within the lungs of *A. muciniphila*-treated mice (**Figures 4D, E**), indicating localized activation of effector T cells. Notably, these changes were lung specific, as no comparable activation was detected in the spleen (data not shown), underscoring that *A. muciniphila* primarily modulates local, rather than systemic, T-cell responses. To evaluate *A. muciniphila*’s impact on SARS-CoV-2-specific responses, we analyzed antigen-specific CD8^+^ T cells using a SARS-CoV-2 tetramer. *A. muciniphila* pre-treatment significantly increased the proportion of activated tissue-resident memory T (T_RM_) cells expressing CD69^+^ and/or CD103^+^ markers among SARS-CoV-2-specific CD8^+^ T cells (**Figures 4F, G**). Although the total numbers of T_RM_ cells showed only marginal increases, their enrichment highlights *A. muciniphila*’s role in promoting SARS-CoV-2-specific T_RM_ cell establishment (**Supplementary Figure S5D**). Importantly, the abundance of SARS-CoV-2-specific CD69^+^ CD103^+^ T_RM_ cells negatively correlated with temperature loss during SARS-CoV2 infection (**Supplementary Figure S6**), suggesting their functional relevance in mitigating infection-induced pathology. These findings collectively indicate that *A. muciniphila* enhances localized T cell responses, promotes T_RM_ cell formation, and supports durable immune memory in the lungs, contributing to improved host resilience against viral infections.

### *Akkermansia muciniphila* enhances cytokine secretion and polyfunctional T-cell responses in the lung

**Figure 5:**
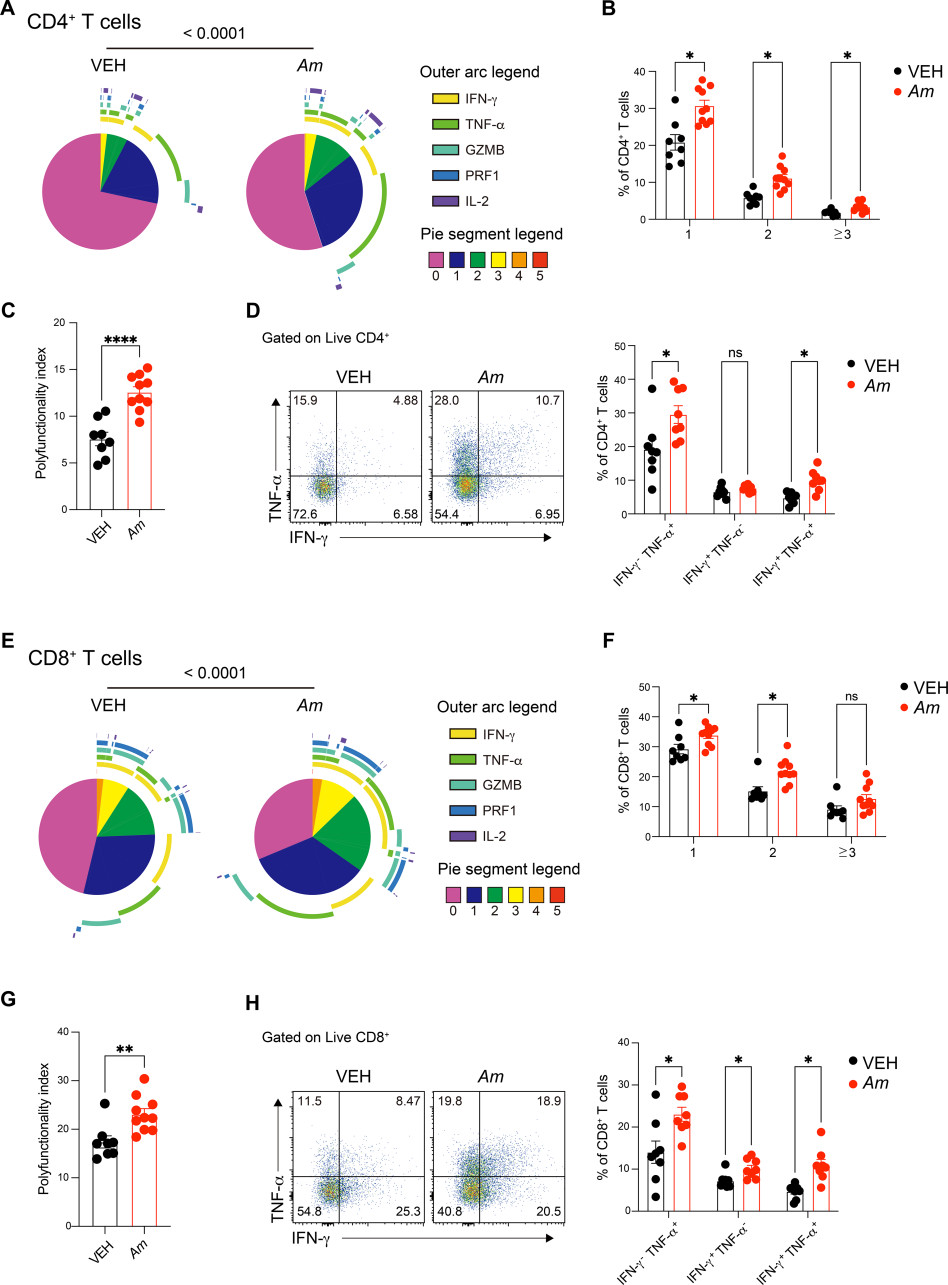
*Akkermansia muciniphila* pre-treatment enhances polyfunctionality and cytokine production in lung CD4^+^ and CD8^+^ T cells. **(A)** Pie charts representing the distribution of cytokine production profiles (IFN-γ, TNF-α, GRANZYME, PERFORIN, and IL-2) in CD4^+^ T cells from the lungs of vehicle-treated and *A. muciniphila*-treated mice. The outer arc represents the type of cytokine, and the pie segments show the number of cytokines secreted by individual CD4^+^ T cells. **(B)** Quantification of the percentage of CD4^+^ T cells producing one or more cytokines. **(C)** Polyfunctionality index of CD4^+^ T cells. **(D)** Bar graphs showing the percentage of CD4^+^ T cells producing individual cytokines (IFN-γ, TNF-α), with representative flow cytometry plots below indicating cytokine-positive CD4^+^ T cells. **(E)** Pie charts showing the distribution of cytokine production profiles in CD8^+^ T cells from vehicle-treated and *A. muciniphila*-treated mice. **(F)** Quantification of the percentage of CD8^+^ T cells producing one or more cytokines. **(G)** Polyfunctionality index of CD8^+^ T cells. **(H)** Bar graphs showing the percentage of CD8^+^ T cells producing individual cytokines (IFN-γ, TNF-α), with representative flow cytometry plots below illustrating cytokine-positive CD8^+^ T cells. Statistical significance values were determined using unpaired *t*-tests. *p < 0.05; **p < 0.01; ****p < 0.0001; ns, not significant.

To assess the functional impact of *A. muciniphila* on lung immune responses, we analyzed cytokine secretion profiles in lung-resident immune cells. While no significant changes were observed in non-T-cell populations (**Supplementary Figures S7A, B**), *A. muciniphila*-treated mice exhibited a marked enhancement in cytokine production by lung-resident T cells compared to vehicle-treated controls (**Figure 5**). Notably, the polyfunctionality index, which reflects the ability of individual T cells to secrete multiple cytokines simultaneously, was significantly elevated in *A. muciniphila*-treated mice (**Figures 5A–C**; **Supplementary Figure S8A**). This enhanced polyfunctionality highlights the increased functional capacity of CD4^+^ T cells, particularly in their antiviral responses. Specifically, secretion of TNF-α and IFN-γ, two key cytokines for antiviral immunity, was significantly upregulated in CD4^+^ T cells following *A. muciniphila* treatment (**Figure 5D**). A similar enhancement was observed in CD8^+^ T cells, with *A. muciniphila*-treated mice displaying significantly higher cytokine secretion and polyfunctional capacity compared to controls (**Figures 5E–G**; **Supplementary Figure S8B**). The cytokine profile of CD8^+^ T cells revealed a notable increase in TNF-α and IFN-γ production (**Figure 5G**), further emphasizing the improved antiviral functionality of these cells. Collectively, our results demonstrate that *A. muciniphila* promotes localized, lung-restricted immune modulation, leading to iBALT formation, T-cell activation, TRM enrichment, and enhanced polyfunctionality. These findings align with clinical observations of gut–lung crosstalk in COVID-19 (6–8) and further highlight *A. muciniphila* as a potential modulator of respiratory immune defense, supporting its therapeutic potential in viral infections.

## Discussion

This study provides novel insights into the gut–lung axis by demonstrating how SARS-CoV-2 infection alters gut microbiota composition and functionality while shaping immune responses in the respiratory system. Our findings reveal that SARS-CoV-2 variants, WA and Omicron, elicit distinct effects on the gut microbiota. WA infection, in particular, led to significant increases in *Akkermansia muciniphila* and predicted functional enrichment in pathways related to mucin degradation and lipid metabolism. Importantly, *A. muciniphila* treatment enhanced lung-specific immune responses, including cytokine production, the formation of inducible bronchus-associated lymphoid tissue (iBALT), and the activation and expansion of T cells including tissue-resident memory T (T_RM_) cells. These results together underscore the critical role of the gut–lung axis in modulating host responses during SARS-CoV-2 infection and highlight *A. muciniphila* as a promising therapeutic target for respiratory viral infections.

A recent K18-hACE2 study directly compared Wuhan and Omicron variant infections and profiled both intestinal (fecal) and lung microbiota using 16S rRNA sequencing across acute time points (47). Similar to our WA versus Omicron comparison, they reported that intestinal community structure differed by variant over time, with the Wuhan variant infection showing a more pronounced dysbiotic shift during the acute phase, whereas Omicron exhibited a more attenuated trajectory with partial recovery at later time points. At the taxonomic level, Kim et al. highlighted variant-associated shifts across multiple intestinal genera (e.g., enrichment of taxa such as *Mucispirillum* in the Wuhan variant group), whereas our longitudinal fecal profiling identified a particularly robust and reproducible enrichment of *Akkermansia muciniphila*—most prominently under WA infection—and we further demonstrate a functional consequence of this enrichment in promoting iBALT formation and TRM expansion in the lung.

SARS-CoV-2 infection disrupts gut microbiota composition through multiple mechanisms. Direct viral invasion of gut epithelial cells, mediated by ACE2 expression, disrupts epithelial integrity, amino acid transport, and antimicrobial peptide production, leading to an inflammatory milieu that favors the growth of opportunistic pathogens like *Enterobacteriaceae* while depleting beneficial taxa, such as SCFA-producing bacteria from *Lachnospiraceae* and *Ruminococcaceae* families (10, 48, 49). Systemic inflammation further exacerbates gut dysbiosis by impairing mucosal immunity and allowing microbial products, including lipopolysaccharides (LPS), to translocate into the bloodstream, amplifying systemic inflammation and cytokine storms, hallmarks of severe COVID-19 (50, 51). These changes are consistent with observed reductions in microbial diversity, depletion of SCFA-producing bacteria, and enrichment of mucin-degrading bacteria, such as *A. muciniphila*.

Interestingly, the role of *A. muciniphila* in SARS-CoV-2 pathophysiology appears to be context dependent. As a mucin degrader, *A. muciniphila* could exacerbate intestinal inflammation by disrupting the mucus barrier. However, it also plays a crucial role in promoting cytokine production, such as IL-17A and IL-22, which are essential for mucosal tissue repair and immune regulation (52, 53). In our study, *A. muciniphila* pre-treatment not only increased cytokine secretion (e.g., IL-4, IL-5, IL-13, IL-17A/F, and IL-22) but also enhanced lung immune responses, particularly the activation and expansion of T cells including SARS-CoV-2 specific T_RM_ cells and the formation of iBALT. T_RM_ cells, marked by CD69 and CD103 expression, are critical for orchestrating localized immune responses and providing long-term protection against viral pathogens (54). Crucially, while excessive Th2 and Th17 responses can be associated with immunopathology (55), the upregulation observed here was accompanied by reduced weight loss and ameliorated lung pathology rather than tissue damage. We propose that this cytokine profile favors a protective response by facilitating iBALT formation which utilizes Th17 signals (56, 57) and supporting epithelial repair via IL-22 (58), thereby combining effective viral containment with enhanced tissue resilience.

The formation of iBALT, observed following *A. muciniphila* treatment, is another key finding of this study. iBALT is an organized lymphoid structure that facilitates antigen presentation and immune coordination in the lung, enabling localized antiviral responses (56, 57, 59). Mechanistically, *A. muciniphila* may enhance T-cell priming and activation through phospholipids that trigger non-canonical TLR2–TLR1 signaling pathways (60). Additionally, *A. muciniphila*-specific T follicular helper (Tfh) cells may support B cell responses, further promoting iBALT development (61, 62). Our findings also suggest that gut-derived T cells, primed in the gut-associated lymphoid tissue (GALT) during SARS-CoV-2 infection, may migrate to the lungs. This migration is likely facilitated by the upregulation of adhesion molecules, such as MAdCAM-1 and VCAM-1, in lung tissue, as confirmed by increased mRNA expression levels during infection (data not shown). These primed T cells contribute to the establishment of T_RM_ cells and iBALT, enhancing antiviral immunity and limiting systemic inflammation.

Beyond the immunophenotypic changes demonstrated in our study, several emerging molecular mechanisms suggest how *Akkermansia muciniphila* may actively orchestrate the lung-specific immune landscape during SARS-CoV-2 infection. First, *A. muciniphila* has been shown to induce antigen-specific CD4^+^ T follicular helper (Tfh) cells and systemic IgG1 responses under homeostatic conditions (61). This capacity to elicit cognate T cell responses may underlie the TRM enrichment and B-cell-associated iBALT formation observed in our SARS-CoV-2 mouse model, suggesting that *A. muciniphila* antigens contribute to localized lymphoid structure formation. In addition, our finding that lung CD8^+^ T cells exhibited enhanced polyfunctionality and increased TRM markers after *A. muciniphila* colonization is consistent with recent reports of *A. muciniphila*–derived outer membrane proteins such as Amuc_1434 and Amuc_1100, promoting CD8^+^ T cell activation by downregulating PD-L1 expression in epithelial cells (63, 64). These proteins may act synergistically with SARS-CoV-2 antigens in shaping robust antiviral CD8^+^ T-cell responses at mucosal sites. Further supporting this, phosphatidylethanolamine a15:0-i15:0 PE, a bioactive lipid uniquely secreted by *A. muciniphila*, activates the TLR2–TLR1 axis to induce TNF-α and IL-6 in dendritic cells (60). In our model, we observed increased Th2 and Th17 cytokines—profiles known to be induced downstream of non-canonical TLR2 signaling—and iBALT structures rich in APCs and T cells. These observations raise the possibility that microbial lipids such as a15:0-i15:0 PE serve as innate adjuvants, priming mucosal immunity and facilitating the spatial organization of iBALT. Lastly, *A. muciniphila*-mediated modulation of tryptophan metabolism and increased levels of indolepropionate, an AhR ligand with known effects on mucosal healing and IL-22 production, may synergize with the IL-22 elevation observed in our model following infection (65). These metabolite-driven pathways likely contribute to the epithelial protection and mucosal resilience that accompany improved disease outcomes. Collectively, these mechanisms suggest that *A. muciniphila* not only colonizes the gut but also programs distal mucosal immunity via a coordinated network of microbial antigens, lipids, enzymes, and metabolites. Our study provides the first *in vivo* evidence that such mechanisms can converge to establish iBALT, enhance TRM differentiation, and amplify localized Th2/Th17 cytokine responses during respiratory viral infection.

While these findings highlight the critical role of *Akkermansia muciniphila* in enhancing immune responses during SARS-CoV-2 infection, several limitations should be acknowledged to guide future research. First, the use of the K18-hACE2 transgenic mouse model, while widely adopted in SARS-CoV-2 studies due to its susceptibility to infection, may not fully replicate the immunological and physiological responses seen in humans (66). Notably, this model overexpresses human ACE2 under a cytokeratin promoter, resulting in neuroinvasion and lethality patterns that differ from most human cases. Therefore, caution is needed when extrapolating findings to clinical contexts. Second, although our study used antibiotic-treated mice as a pseudo–germ-free model, this approach lacks the immunological and developmental naivety of true germ-free animals. Antibiotic treatment can cause incomplete microbiota depletion and off-target effects on immune priming, potentially affecting the colonization efficiency and immunomodulatory functions of *A. muciniphila*. Third, while we identified robust immune phenotypes—such as iBALT formation and TRM enrichment—the detailed molecular and cellular mechanisms underlying *A. muciniphila*-driven immunity remain to be elucidated. Future work should focus on dissecting the roles of specific bacterial metabolites, membrane proteins (e.g., Amuc_1100, Amuc_1434), and host signaling pathways such as TLR2-CREB, PD-L1, and AhR in mediating these immune effects.

In conclusion, this study provides compelling evidence for the critical role of *Akkermansia muciniphila* in modulating mucosal immunity during SARS-CoV-2 infection via the gut–lung axis. Notably, the consistent enrichment of *A. muciniphila* observed in both SARS-CoV-2-infected mice and human COVID-19 patients suggests that this may represent a host-intrinsic adaptive response to reinforce mucosal defenses. Rather than being a mere byproduct of dysbiosis, the expansion of *A. muciniphila* may serve as a natural immunological strategy to prime the respiratory immune landscape through gut–lung axis modulation. Using a K18-hACE2 mouse model, we demonstrate that *A. muciniphila* colonization enhances lung-specific immune responses by promoting localized cytokine production, enriching tissue-resident memory T cells, and inducing the formation of inducible bronchus-associated lymphoid tissue (iBALT). These effects collectively contributed to reduced disease severity and improved immune containment of viral pathology, without provoking systemic inflammation. The immunomodulatory actions of *A. muciniphila* are likely mediated by a diverse repertoire of microbial antigens, membrane proteins, and metabolites, including TLR2-activating phospholipids and IL-10-inducing enzymes, which together orchestrate both innate and adaptive antiviral defenses. Importantly, the implications of this work may extend beyond acute COVID-19. Emerging evidence links long COVID—characterized by prolonged fatigue, cognitive dysfunction, and persistent inflammation—with microbiota dysbiosis and chronic immune dysregulation (67). Given *A. muciniphila*’s demonstrated capacity to resolve inflammation and restore mucosal immune balance, its administration may offer therapeutic benefit in ameliorating post-viral sequelae. Thus, *A. muciniphila* represents a promising microbiota-derived intervention for both acute and chronic respiratory disease states. Future work should prioritize the mechanistic dissection of host–microbe signaling pathways and advance clinical trials to evaluate the safety, efficacy, and translatability of *A. muciniphila*-based therapies in diverse patient populations. By leveraging the microbial control of immune tone, we may open new avenues for microbiome-driven treatment strategies in viral immunopathology. 

## Data Availability

The datasets presented in this study can be found in online repositories. The names of the repository/repositories and accession number(s) can be found in the article/**Supplementary Material**.

## Supplementary material

Supplementary Figure 1 Gut microbiome alterations in aged mice by SARS-CoV-2 infection. **(A)** Study design for WA infection in aged mice. Fecal samples were collected based on the indicated timeline (*n* = 5–10 mice per group). **(B)** Alpha diversity (observed features and Shannon index) of aged mice after WA infection. **(C)** Taxa bar plot of feces microbiome in WA-infected mice (family level). **(D)** Volcano plot of edgeR results of WA-infected aged mice to test enriched taxa in 0 and 5 dpi.

Supplementary Figure 2 *Akkermansia* spp. is associated with COVID-19-mediated pathogenic phenotype. **(A)** Heatmap of Pearson’s correlation coefficients between bacterial taxa and temperature and weight change before (0 dpi) and after (5 dpi) WA infection. The color scale represents the strength and direction of the correlation, with blue indicating negative correlations and red indicating positive correlations. Asterisks denote statistically significant correlations (**p* < 0.05; ***p* < 0.01; ****p* < 0.001). **(B)** Scatter plot showing the positive correlation between *Akkermansiaceae* (*Akkermansia*) abundance and temperature change (ΔTemperature). The solid line represents the linear regression fit, with the shaded area showing the 95% confidence interval. The Pearson correlation coefficient (*R*) and associated *p*-value are displayed. **(C)** Scatter plot illustrating the positive correlation between *Akkermansiaceae* (*Akkermansia*) abundance and weight change (ΔWeight). The solid line shows the linear regression fit, and the shaded area represents the 95% confidence interval. The Pearson correlation coefficient (R) and the corresponding *p*-value are indicated. **(D)** Distribution of *Akkermansia* carriers (red) and non-carriers (gray) in control (CON) and COVID-19 (COV) groups across five independent studies. The stacked bar graph illustrates the sample numbers for each category. **(E)** Relative abundance (%) of *Akkermansia* in healthy controls (CON) and COVID-19 patients (COV) across all studies. Data points represent individual samples. Statistical significance was determined using Mann–Whitney test (***p* < 0.01). **(F)** Relative abundance (%) of *Akkermansia* stratified by COVID-19 disease severity. Samples were classified as healthy controls (CON) or COVID-19 patients with severity I (asymptomatic/mild), severity II (moderate), or severity III (severe/critical). Statistical analysis was performed using Kruskal–Wallis test (**p* < 0.05).

Supplementary Figure 3 Evaluation of immune responses in a SARS-CoV-2 infection model treated with *Akkermansia muciniphila* or vehicle control. **(A)** Relative abundance of *A. muciniphila* rRNA in total 16S rRNA from fecal samples before ABX treatment and after vehicle or *A. muciniphila* treatment. Statistical significance was calculated using one-way ANOVA. ***p* < 0.01; ****p* < 0.001. **(B)** Viral load in the lungs, quantified as log PFU/g in *A. muciniphila*- and vehicle-treated groups. **(C)** *In situ* hybridization analysis of human ACE2 (hACE2) expression in the lung tissues of K18-hACE2 mice with or without *Akkermansia muciniphila* treatment. **(D)** Heatmap of serum cytokine profiles showing log2 fold change of cytokine levels across mock-, vehicle-, and *A. muciniphila*-treated groups. **(E)** Percentage of F4/80^+^ macrophages in the lung tissue. **(F)** Percentage of Ly6G/Ly6C^+^ neutrophils in the lung tissue. Statistical significance was determined using unpaired *t*-tests. ns, not significant.

Supplementary Figure 4 Gating strategies. Gating strategies for **(A)** total immune cell, **(B)** T cell, and **(C)** cytokine panel.

Supplementary Figure 5 Comparison of immune cell populations in the lungs of *A. muciniphila*- and vehicle-treated mice. **(A)** Proportions and **(B)** absolute numbers of immune cell populations, including NK cells, γδ T cells, neutrophils, monocytes, dendritic cells, conventional T cells, regulatory T cells, and CD8^+^ T cells. **(C)** Proportion (left) and number (right) of CD44^+^CD62L^-^ effector CD8^+^ T cells in the lung tissue. **(D)** Proportion (left) and number (right) of SARS-CoV-2-specific tetramer^+^ CD8^+^ T cells in the lung tissue. Statistical significance was determined using unpaired *t*-tests. ns, not significant.

Supplementary Figure 6 Correlation analysis of T-cell subsets with SARS-CoV2-induced pathological changes. **(A)** Scatter plot illustrating the negative correlation between Tet^+^ CD69^+^ CD103^+^ T-cell abundance and temperature change (ΔTemperature). **(B)** Scatter plot showing the negative correlation between Tet^+^ CD69^+^ CD103^-^ T cell abundance and temperature change (ΔTemperature). The solid line indicates the linear regression fit, and the shaded area represents the 95% confidence interval. The Pearson correlation coefficient (R) and associated *p*-value are provided.

Supplementary Figure 7 Comparison of cytokine production profiles in immune cells from the lungs of *A. muciniphila*-pretreated and vehicle-treated mice. **(A)** Proportion and **(B)** absolute numbers of cytokine-expressing cells, including IL-2, PERFORIN, GRANZYME B, TNF-α, and IFN-γ, across various immune cell populations such as B cells, NK cells, γδ T cells, neutrophils, monocytes, macrophages, and dendritic cells. Statistical significance was determined using unpaired *t*-tests. **p* < 0.05; ns, not significant.

Supplementary Figure 8 Analysis of cytokine production in lung CD4^+^ and CD8^+^ T cells from *A. muciniphila*- and vehicle-treated groups. **(A)** Quantification and comparison of cytokine production combinations (IL-2, PERFORIN, GRANZYME B, TNF-α, and IFN-γ) in CD4^+^ T cells between vehicle-treated and *A. muciniphila*-treated mice. **(B)** Quantification and comparison of cytokine production combinations (IL-2, PERFORIN, GRANZYME B, TNF-α, and IFN-γ) in CD8^+^ T cells between vehicle-treated and *A. muciniphila*-treated mice. Statistical significance was determined using unpaired *t*-tests. **p* < 0.05; ***p* < 0.01; ****p* < 0.001.

Supplementary Table 1 Antibody information.
