# Supplementary material for: Akkermansia muciniphila primes lung-resident antiviral immunity via the gut–lung axis during SARS-CoV-2 infection
Source: Front Immunol. 2026 Feb 27;17:1762843. doi: 10.3389/fimmu.2026.1762843 (PMC12982362; doi:10.3389/fimmu.2026.1762843)

Supplementary figure 1.

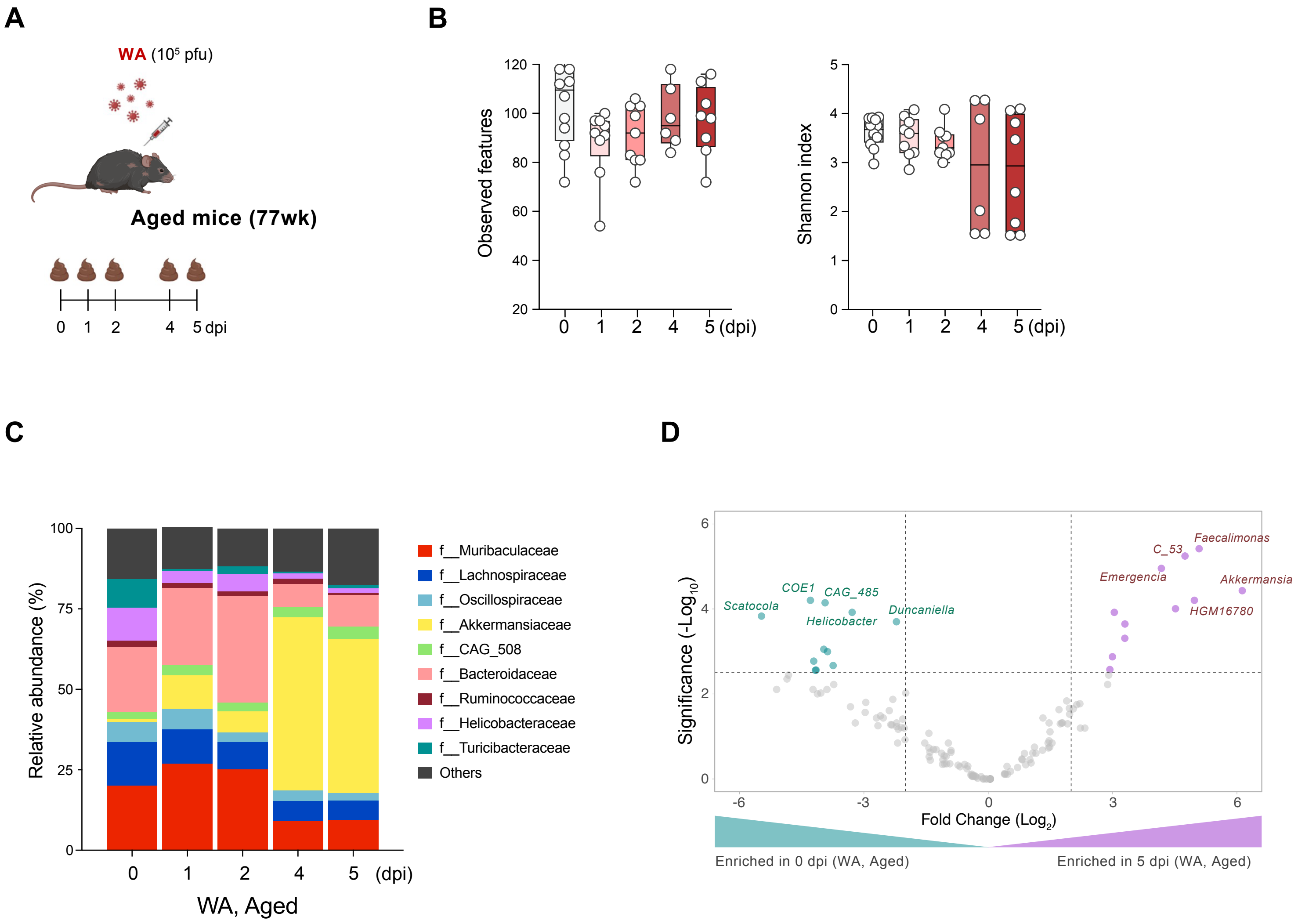

Supplementary figure 2.

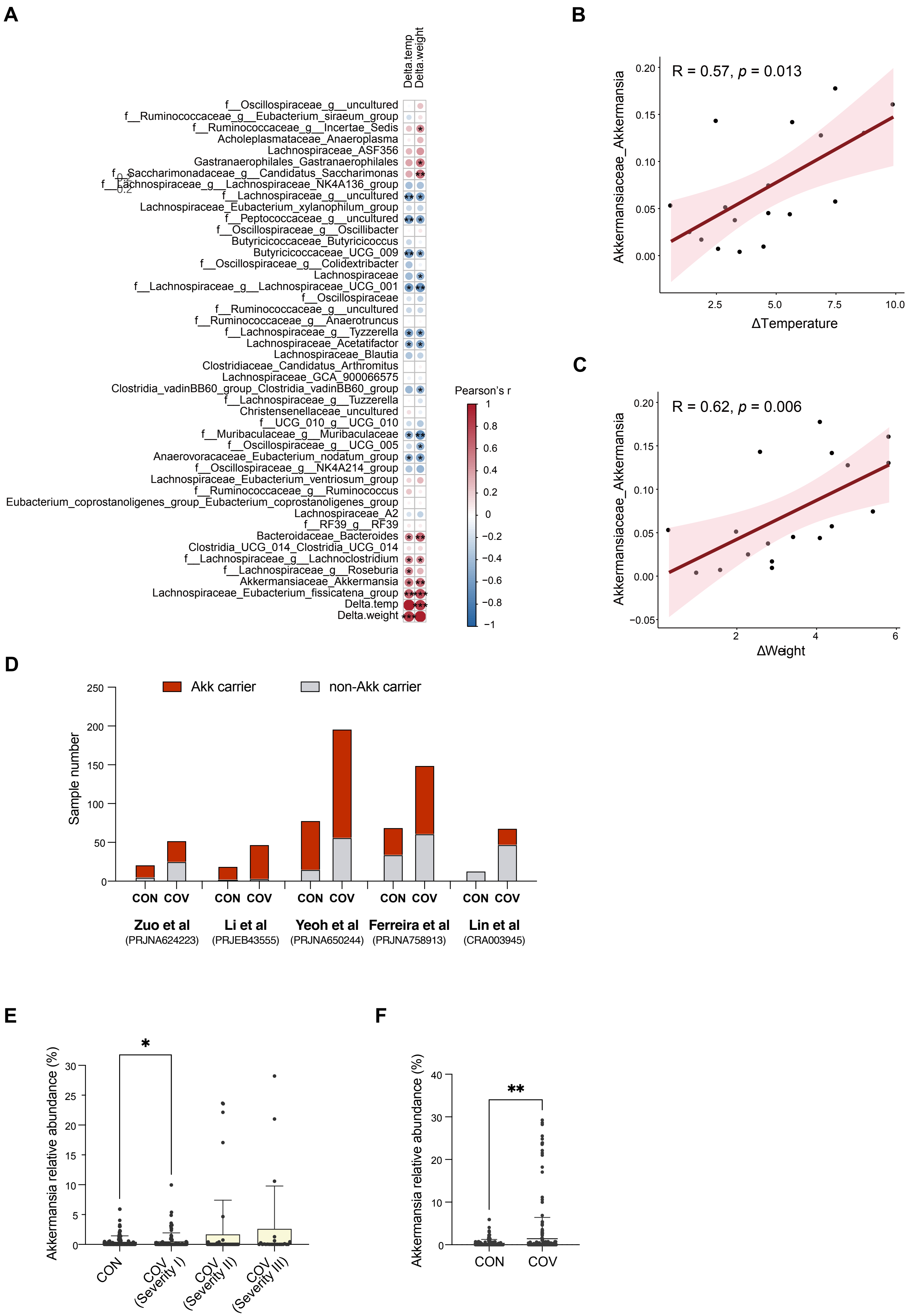

Supplementary figure 4.

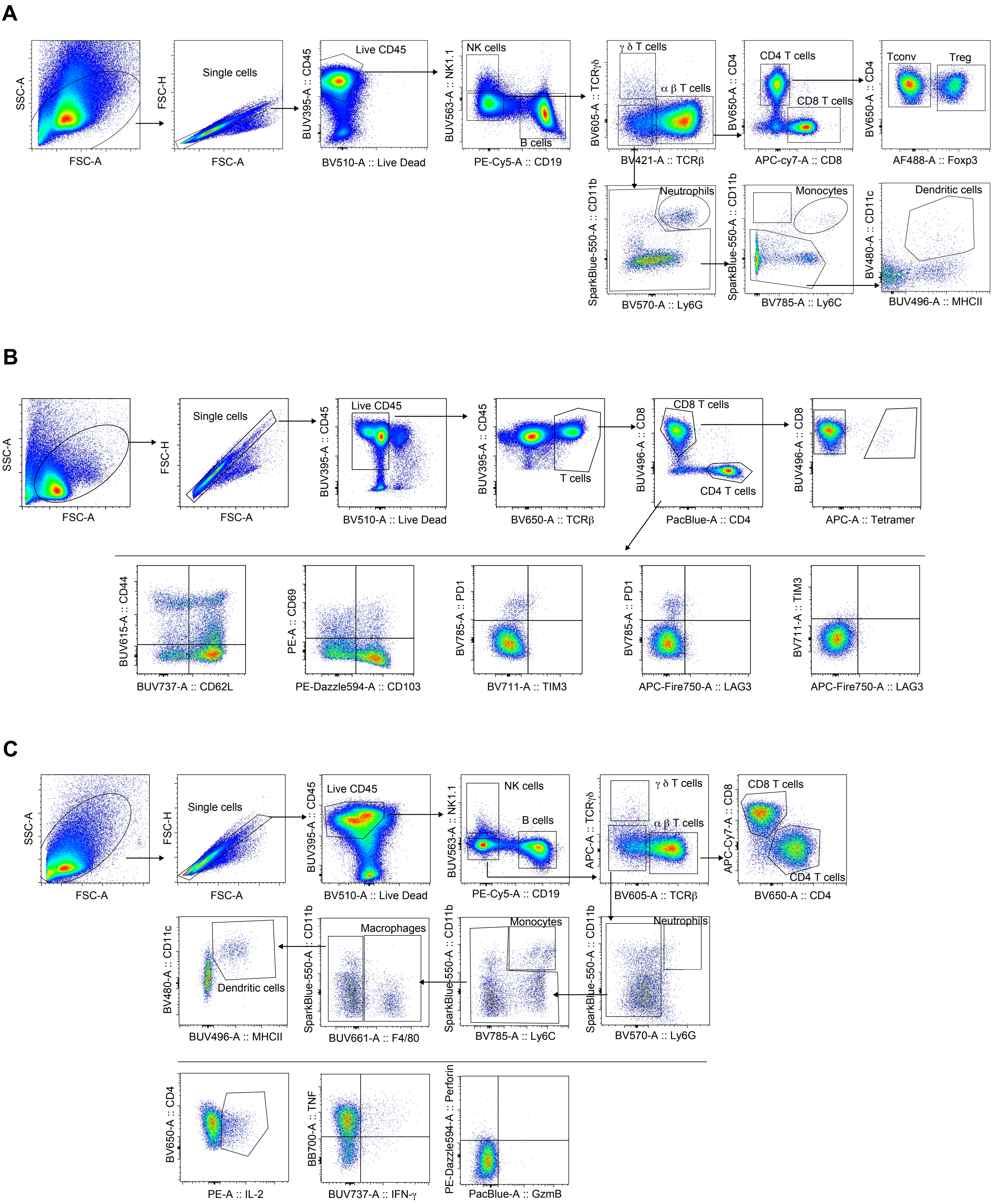

Supplementary figure 5.

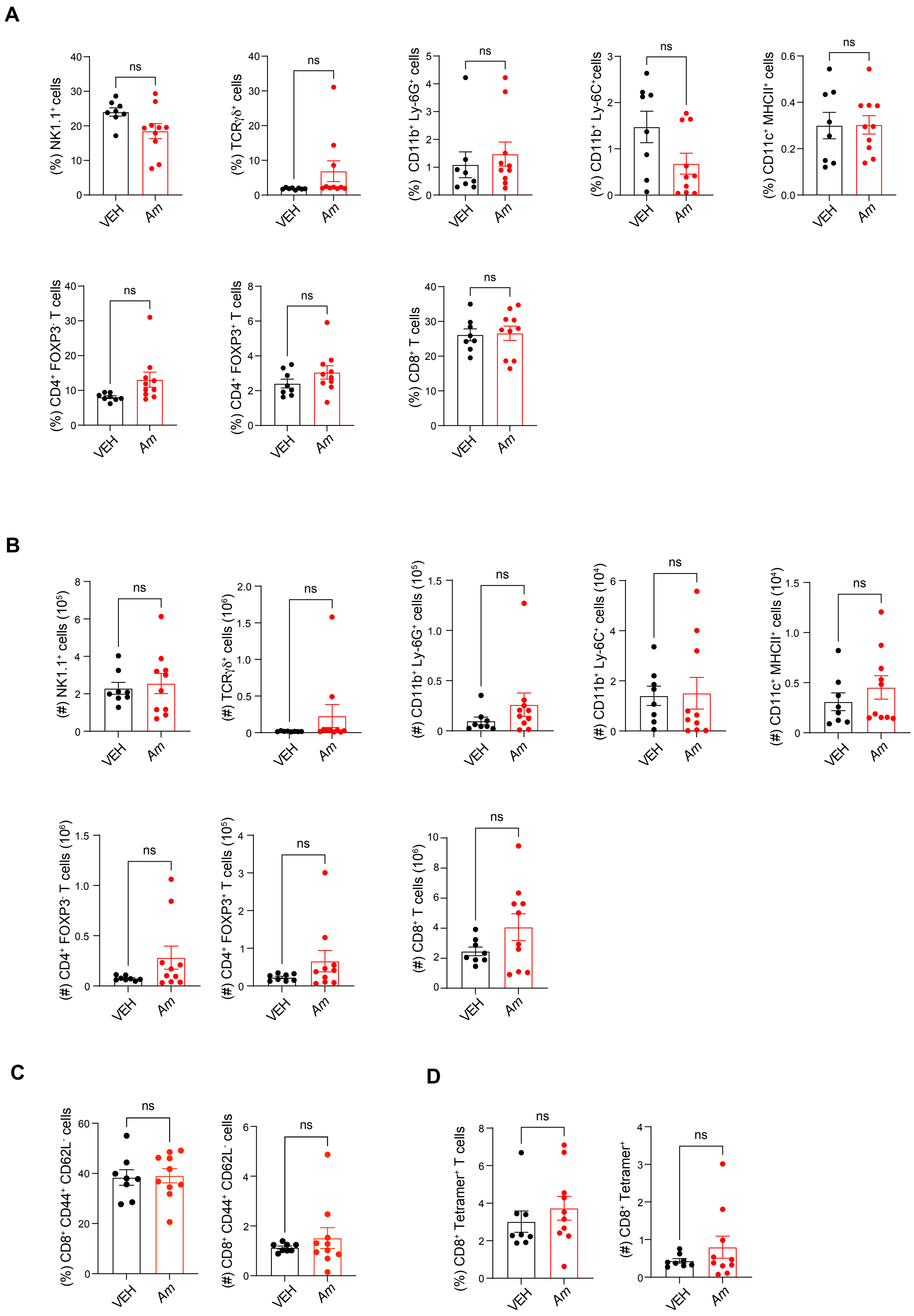

Supplementary figure 6.

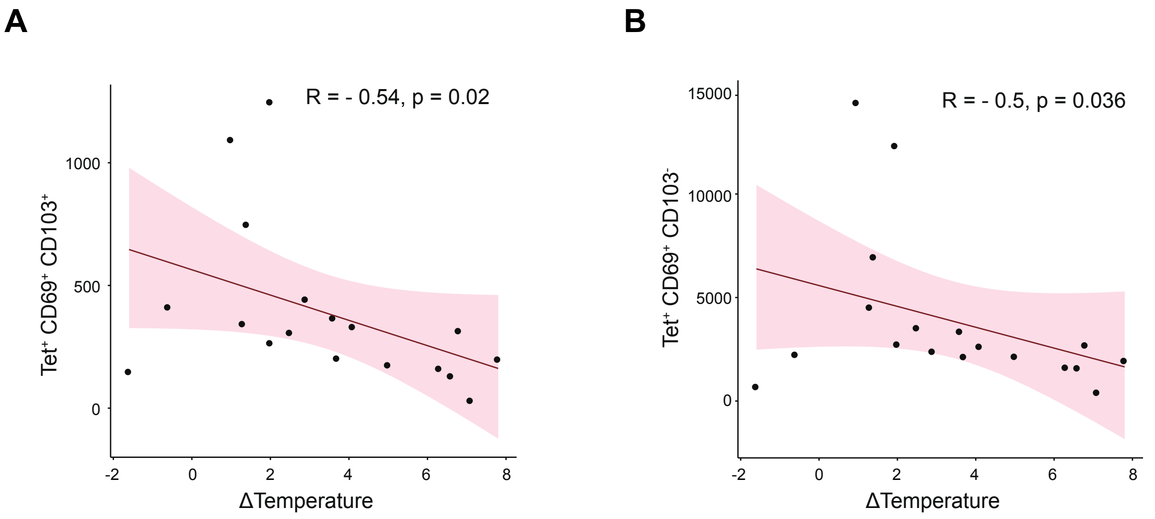

Supplementary figure 7.

A

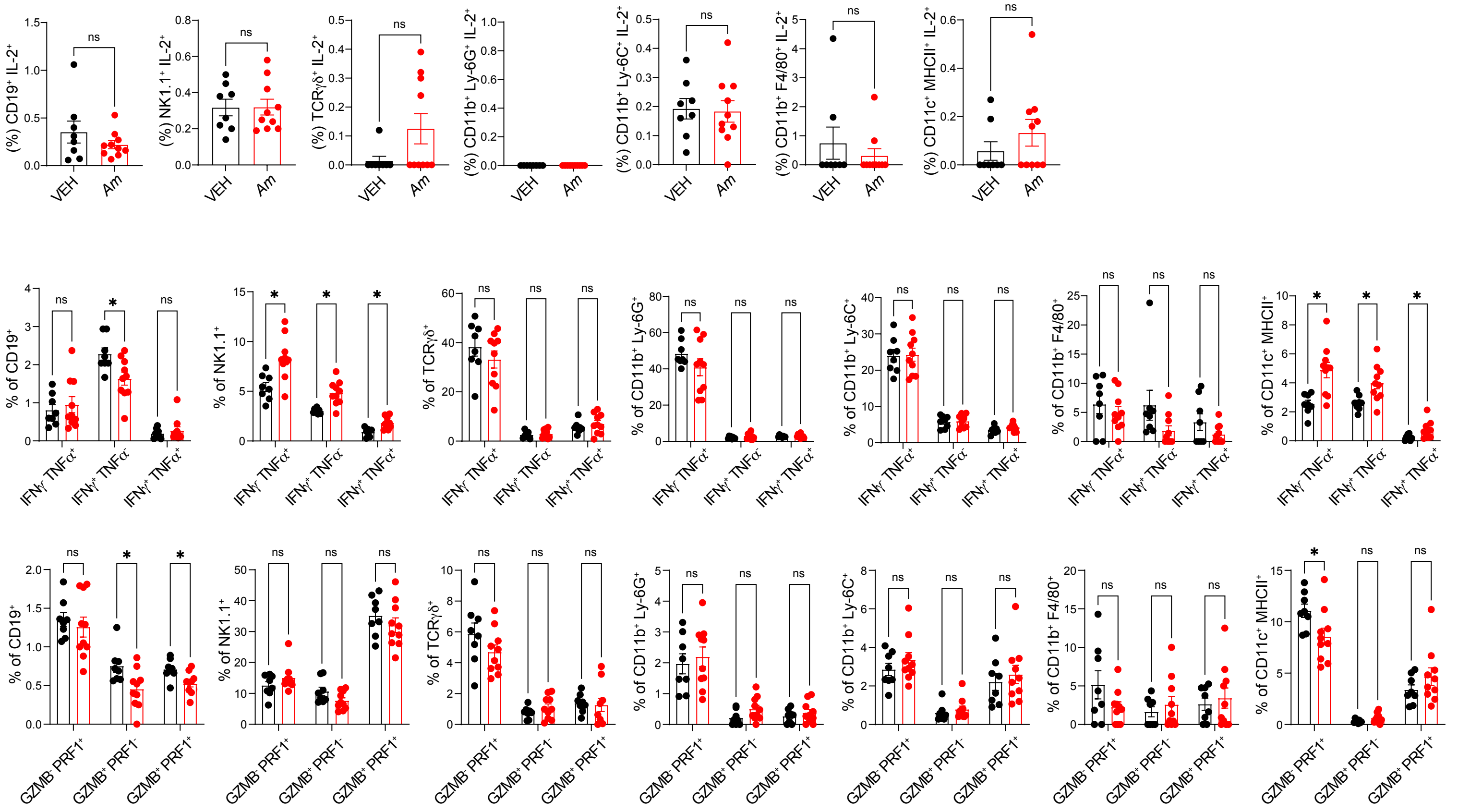

B

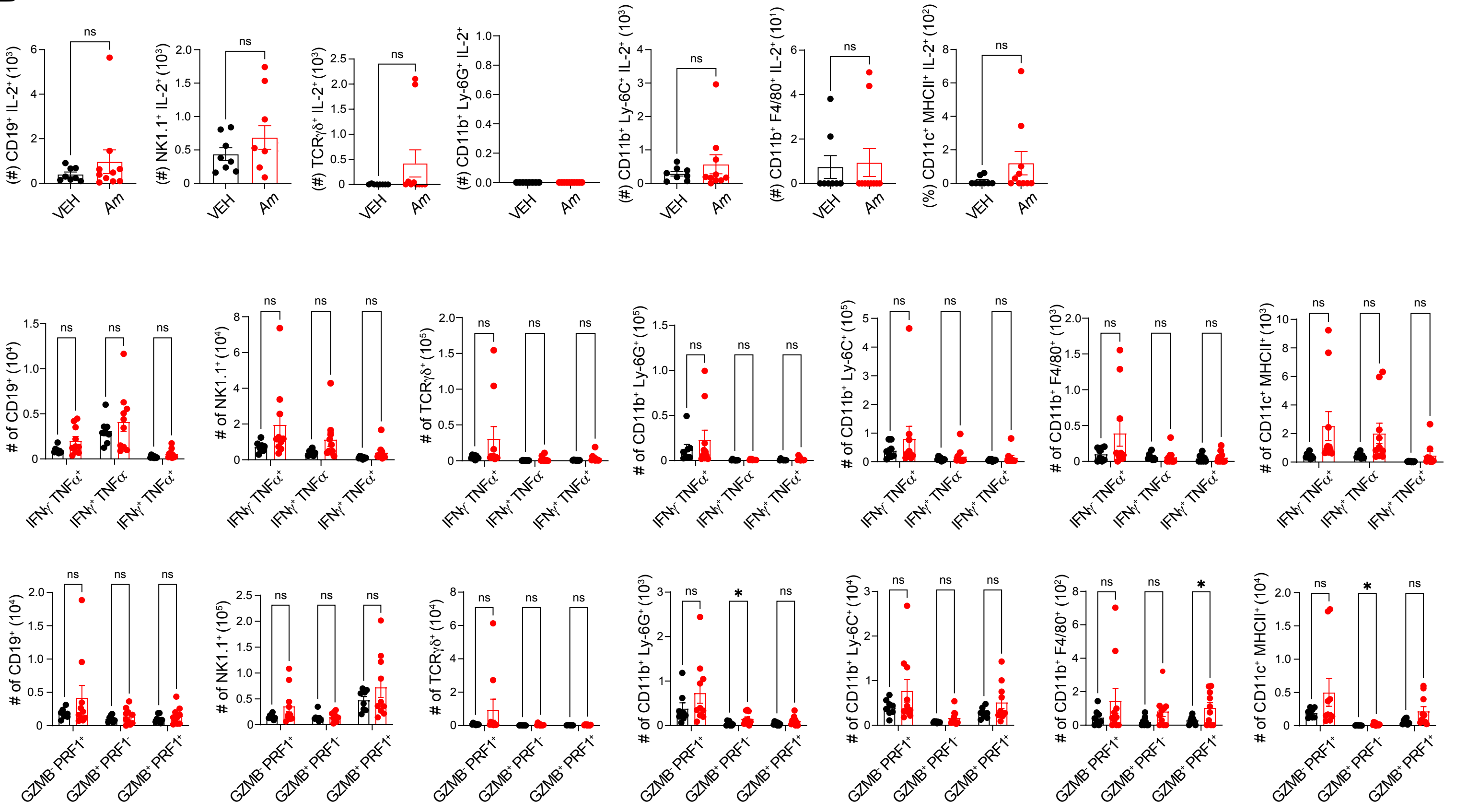

Supplementary figure 8.

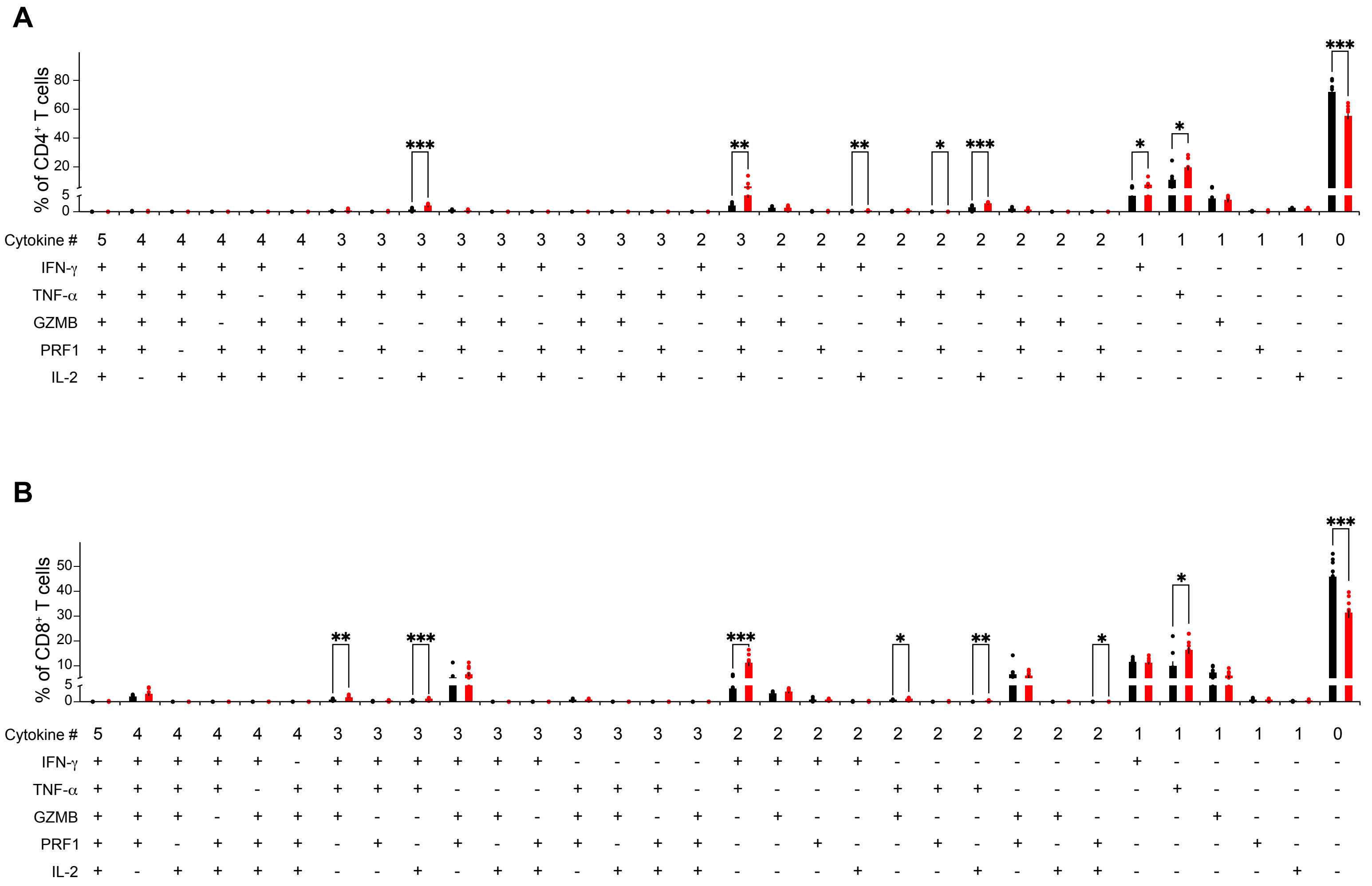

Supplement: Supplementary Figure 1 — Gut microbiome alterations in aged mice by SARS-CoV-2 infection. (A) Study design for WA infection in aged mice. Fecal samples were collected based on the indicated timeline (n = 5–10 mice per group). (B) Alpha diversity (observed features and Shannon index) of aged mice after WA infection. (C) Taxa bar plot of feces microbiome in WA-infected mice (family level). (D) Volcano plot of edgeR results of WA-infected aged mice to test enriched taxa in 0 and 5 dpi. [file Image1.pdf]
